# Effect of Polyvinyl Alcohol Ligands on Supported Gold Nano-Catalysts: Morphological and Kinetics Studies

**DOI:** 10.3390/nano11040879

**Published:** 2021-03-30

**Authors:** Stefano Scurti, Eleonora Monti, Elena Rodríguez-Aguado, Daniele Caretti, Juan Antonio Cecilia, Nikolaos Dimitratos

**Affiliations:** 1 Industrial Chemistry “Toso Montanari” Department, University of Bologna, Viale Risorgimento 4, 40126 Bologna, Italy; stefano.scurti2@unibo.it (S.S.); eleonora.monti8@unibo.it (E.M.); 2 Departamento de Química Inorgánica, Cristalografía y Mineralogía (Unidad Asociada al ICP-CSIC), Facultad de Ciencias, Universidad de Málaga, Campus de Teatinos, 29071 Málaga, Spain; aguadoelena5@gmail.com (E.R.-A.); jacecilia@uma.es (J.A.C.)

**Keywords:** gold nanoparticles, hydrogenation of 4-nitrophenol, polyvinyl alcohol ligands

## Abstract

The effect of polyvinyl alcohol (PVA) stabilizers and gold nanoparticles supported on active carbon (AuNPs/AC) was investigated in this article. Polymers with different molecular weights and hydrolysis degrees have been synthesized and used, like the stabilizing agent of Au nano-catalysts obtained by the sol-immobilization method. The reduction of 4-nitrophenol with NaBH_4_ has been used as a model reaction to investigate the catalytic activity of synthesized Au/AC catalysts. In addition, we report several characterization techniques such as ultraviolet-visible spectroscopy (UV-Vis), dynamic light scattering (DLS), X-ray diffraction (XRD), transmission electron microscopy (TEM), and X-ray photoelectron spectroscopy (XPS) in order to correlate the properties of the polymer with the metal nanoparticle size and the catalytic activity. A volcano plot was observed linking the catalytic performance with hydrolysis degree and the maximum of the curve was identified at a value of 60%. The Au:PVA-60 weight ratio was changed in order to explain how the amount of the polymer can influence catalytic properties. The effect of nitroaromatic ring substituents on the catalytic mechanism was examined by the Hammett theory. Moreover, the reusability of the catalyst was investigated, with little to no decrease in activity observed over five catalytic cycles. Morphological and kinetic studies reported in this paper reveal the effect of the PVA polymeric stabilizer properties on the size and catalytic activity of supported gold nanoparticles.

## 1. Introduction

Metal nanoparticles of different sizes, shapes, and compositions are of high interest in fields such as catalysis, nanomedicine, and pollution remediation [1,2,3,4,5,6]. The decrease in the nanoparticle size evidences a variation of their properties, due to the resulting higher surface area and the quantum confinement of the electronic state. The high surface energy makes nanoparticles unstable compared to the corresponding macroscopic materials. Therefore, to avoid the phenomenon of nanoparticle aggregation, different stabilization strategies have been utilized, which aim to counterbalance Van der Waals’s attractive forces [7].

The properties resulting from the combination of dimensional and quantum effects allowed the use of gold nanoparticles (AuNPs) in catalysis, which is in contrast with bulk gold that has a long-standing reputation of being an inert metal. In 1980, Haruta et al. [8,9] were the first to recognize the reactivity of these systems, using them in the oxidation of carbon monoxide at low temperatures. From the work by Haruta and Hutchings, a variety of catalytic applications have been studied in the area of catalysts, such as the oxidation of alcohols and polyols [10,11], the hydrogenation of acetylene or unsaturated aldehydes and acids [12], the synthesis of hydrogen peroxide [13], carbon-carbon coupling reactions [14], and the catalytic and photocatalytic decomposition of organic compounds [15]. Gold nanoparticles may potentially be useful for removing organic pollutants, such as toxic halogenated organic compounds [16], pesticides (endosulfan, malathion, and chlorpyrifos) [17,18], DNAPL, and LNAPL (dense and light nonaqueous phase liquids, respectively) [19,20] and nitro compounds [21]. 

The reduction of nitroaromatic compounds to functionalized anilines has high industrial relevance due to their applications as intermediates in organic synthesis and their pollutant nature. An interesting example is the catalytic reduction of 4-nitrophenol (4-NP) to 4-aminophenol (4-AP). The Environmental Protection Agency (EPA) has defined this compound as a toxic substance to human health [22]. Despite its toxicity, it is widely used at the industrial level for the realization of pesticides, dyes, and synthetic drugs. The current industrial technique to convert 4-nitrophenol involves either the Bechamp reduction reaction or catalytic hydrogenation [23,24]. A large amount of sludge is generated as waste using these methods to eliminate this product. Innovative strategies were developed able to convert 4-nitrophenol to higher value-added chemicals, through catalytic reduction of the nitro group by gold nanoparticles [25,26]. Moreover, the 4-nitrophenol reduction to 4-aminophenol (4-AP) by NaBH_4_ can be considered as a model reaction for the evaluation of its catalytic activity. The reaction occurs at room temperature and ambient pressure, without the formation of side-products, and the conversion can be easily monitored by UV-visible spectroscopy. The reaction presents a high activation barrier due to repulsive forces between 4-nitrophenolate anion and the hydride used as a reducing agent. In the absence of a catalyst, kinetics is extremely slow, this allows making assessments on the real catalytic efficiency of supported metal nanoparticles [27,28]. Several research groups investigated the possible mechanism of the 4-nitrophenol reduction reaction catalyzed by AuNPs. Fountoulaki et al. proposed a catalytic mechanism to explain the reduction of 4-NP, through the study of different para-substituents on the nitroarene ring [29]. The study of Wunder et al. described the interaction of hydrogen resulting from the decomposition of NaBH_4_ on the surface of the metal catalyst and the simultaneous adsorption of 4-nitrophenol molecules by a Langmuir–⁠Hinshelwood mechanism [30]. Moreover, Chiu et al. used computational simulations to predict the catalytically active faces of gold nanoclusters, resulting in the face (111) being more active than the others [31].

Several studies have evidenced the importance of polymeric stabilizers on the catalytic activity of metal-supported nanoparticles in terms of slowing down the electron transfer kinetics in the redox catalysis mechanism on the surface of NPs. Recent works have proposed a different interpretation of the role of polymeric ligands in nano-catalysis. They have used polymer/NP hybrid nanomaterials in order to control the binding of the reagent on the surface and, consequently, on the conversion and selectivity of the reaction [32,33,34,35]. Zhang et al. have described how the use of N-heterocycle carbene (NHC)-based polymeric ligands could enhance the catalytic activity of AuNPs compared to the same catalyst stabilized with thiolate polymers [33]. Yoskamtorn et al. observed, in aerobic oxidation of benzyl alcohol, that the gold nanoclusters fully covered by thiolates ligands were inert, owing to the site-blocking effect [36]. Wu et al. showed the steric effect of the amine ligands length on the cinnamaldehyde hydrogenation selectivity using Pt_3_Co nanocrystals [37]. Chen’s group reported the effect of ligand to modulate the surface charge density of platinum nanowires to tune the hydrogenation selectivity of nitroaromatic compounds [38].

Polyvinyl alcohol (PVA) is a commercially available polymer that is hydro-soluble, biocompatible, has low toxicity, and is slowly biodegradable [39,40]. Moreover, it has shown a stabilizing effect for colloidal solutions prepared in water media [40]. For these reasons, different studies have investigated the role of PVA as steric stabilizers for the synthesis of mono-metallic and bimetallic nanoparticles [41,42,43,44]. Although it is widely used in nano-catalysts preparation methods, few studies have reported the use of customized synthetic polyvinyl alcohol instead of commercial PVA. The development of a synthetic strategy to obtain PVA with different properties in terms of molecular weight and hydrolysis degree was studied in this work.

In addition, we have investigated the correlation between the molecular structures of different PVA ligands with the morphology and catalytic activity of gold nanoparticles supported on activated carbon (AuNPs/AC) in order to understand the mechanism and catalytic activity of gold colloidal nanoparticles for redox reactions. The influence of several experimental parameters, such as the molecular weight and the hydrolysis degree of the polymer ligands, on the morphology of the final catalysts, their stability, and catalytic activity in terms of conversion, selectivity, and availability of active sites on the nanoparticles’ surface, was also evaluated. The catalytic activity of the AuNPs/AC system was studied for the catalytic reduction of 4-NP as the chosen reaction. The effect of PVA on the catalytic activity of AuNP was elucidated using a combination of characterization techniques to connect morphological features to the catalytic activity of these systems, in order to demonstrate the active role of PVA stabilizers in the catalytic mechanism.

## 2. Materials and Methods

### 2.1. Materials

2-2′-Azo-bis-isobutyronitrile (AIBN), vinyl acetate (>99%), tetrachlorauric acid (HAuCl_4_·3H_2_O), sodium borohydride (NaBH_4_, 99%), activated carbon NORIT SX1G, sulfuric acid (H_2_SO_4_, 96%), 4-nitrophenol (>99%), 3-nitrophenol (>99%), 2-nitrophenol (>99%), polyvinyl alcohol 88 (Mw: 13,000–23,000, 88–89% hydrolyzed), polyvinyl alcohol 2 (Mw: 13,000–23,000, 98–99% hydrolyzed), polyvinyl alcohol 3 (Mw: 31,000–50,000, 98–99% hydrolyzed), and polyvinyl alcohol 4 (Mw: 146,000–186,000, 98–99% hydrolyzed) were purchased from Sigma Aldrich (Italy). The other polymers were properly synthesized in the laboratory and experimental protocols are presented below. The solvents were used without further processes of purification.

### 2.2. Synthesis of Polyvinyl Alcohol (PVA)

The polymers were synthesized using the experimental methodology reported by Garaeva et al. [45]. Following the experimental protocol, the polymerization reaction of vinyl acetate was carried out in a three-necked flask placed under a nitrogen atmosphere. The polymer was synthesized by dissolving 10 mL of monomer (0.1 mol) in 10 mL of solvent prepared according to the ratios described in Table 1. After heating the solution up to 68 °C, azo-bis-isobutyronitrile (AIBN) was added as an initiator in a quantity equal to 0.1% mol/mol. The reaction was stirred for 90 min. The next step was to cool down the mixture to room temperature and to drain in a solution of ethyl ether. The polymer was filtered and washed several times with ethyl ether. The polyvinyl-acetate (PVAc) was characterized by nuclear magnetic resonance (NMR), Infrared (IR) spectroscopy, and gel permeation chromatography (GPC) (see Figure S1a,b and Table S1).

The direct saponification reaction of polyvinyl-acetate was performed in a 100 mL flask, in which 0.3 g PVAc was added in 8 mL of acetone. The mixture was stirred at 50 °C to promote the solubilization of the polymer. Once dissolved, NaOH 1 M in methanol was added (1:1 mol/mol). The reaction was carried out for 1 h under reflux conditions. Then, the mixture was filtered and the methanol was evaporated. The product was dried in an oven at 70 °C for 2 h. The polyvinyl alcohol (PVA) was characterized by nuclear magnetic resonance (NMR) and IR spectroscopy (see Figures S2 and S3).

### 2.3. Catalyst Preparation

A colloidal gold solution was prepared by dissolving 0.76 g (1.9 mmol) of HAuCl_4_·3H_2_O in 200 mL of distilled H_2_O. Then 5.5 mL were taken from the initial solution and diluted to 385 mL of distilled H_2_O, to which was added a volume of 1% *w/w* polymer aqueous solution to obtain the desired Au:PVA weight ratio. After 3 min, a freshly prepared aqueous solution of NaBH_4_ (Au:NaBH_4_ = 1:5 mol/mol) was added. A red Au^0^ sol was immediately formed. The colloidal solution was stirred for 30 min and, at the end, the Au colloidal nanoparticles were immobilized by adding the activated carbon (AC) under vigorous stirring. The solution was acidified at pH 2 by using sulfuric acid. The amount of support was calculated in order to have a nominal metal loading of 1 wt.%. The mixture was stirred for 1 h at room temperature. Then, the catalyst was filtered using a Buchner funnel and washed several times with distilled water to remove ionic species and mother liquors, until a neutral pH was reached. After drying overnight at room temperature, the solids were dried at 80 °C in an oven for 4 h in static air conditions. The colloidal solution was characterized by means of UV-visible spectroscopy and dynamic light scattering (DLS). The supported catalysts were characterized using X-ray photoelectron spectroscopy (XPS), transmission electron microscopy (TEM), and X-ray diffraction (XRD).

### 2.4. Characterization

All the polymers were accurately characterized. The PVAc and PVA structures were confirmed by ^1^H and ^13^C NMR spectroscopy, by using Varian “Mercury 400” and “Mercury 600” spectrometers, operating at 400 and 600 MHz, respectively. TMS was used as an internal reference standard. The polymers were characterized by Fourier transform-infrared spectroscopy (FT-IR), acquiring the spectra through an ATR-IR Bruker Alpha I spectrometer. The molecular weights of the polymers were determined by gel permeation chromatography (GPC). The analyses were carried out using two 300 mm 5MXL columns with different ranges of molecular weights that they are able to assess. The samples were solubilized in THF and the analysis was performed with a flow rate of 1 mL/min at 20 °C. The calibration of the column was carried out with monodisperse polystyrene standards.

Colloidal solutions were analyzed by dynamic light scattering (DLS) measures, registered by using a Malvern Zetasizer Nano ZS instrument. The particle size analysis was carried out using a standard polystyrene cell at 25 °C, while for the zeta potential analysis a capillary polycarbonate cell equipped with electrodes at 25 °C was employed. The poly-dispersity index (PI) was studied by DLS analysis, it was given on a scale from 0 to 1, where the mono-dispersity was represented of 0 value [46]. UV-visible spectra were recorded on an Agilent Cary 3500 UV-Vis Spectrometer. Transmission electron microscopy (TEM) images were obtained using a TEM Talos F200X instrument. TEM images were processed by using the ImageJ in order to determine average particle size and particle size distribution. X-ray photoelectron spectroscopy (XPS) spectra were recorded on a Physical Electronic spectrometer (PHI Versa Probe II) using monochromatic Al Kα radiation (52.8 W, 15 kV, 1486.6 eV) and a dual-beam charge neutralizer for analyzing the core-level signals of the elements of interest with a hemispherical multichannel detector. The samples’ spectra were recorded with a constant pass energy value at 29.35 eV and a beam diameter of 100 µm. The energy scale was calibrated using Cu 2p_3/2_, Ag 3d_5/2_, and Au 4f_7/2_ photoelectron lines at 932.7, 368.2, and 83.95 eV, respectively. The X-ray photoelectron spectra obtained were analyzed using PHI SmartSoft software and processed using the MultiPak 9.6.0.15 package. The binding energy values were referenced to C 1s signal at 284.5 eV. Shirley-type background and Gauss–⁠Lorentz curves were used to determine the binding energies. Atomic concentration percentages of the characteristic elements were determined considering the corresponding area sensitivity factor for the different measured spectral regions. Powder X-ray diffraction (XRD) patterns were recorded on a PANalytical X’PertPRO X-ray diffractometer. A Cu radiation source (λ= 1.54 Å) was utilized, and diffraction patterns were recorded between 10–80° 2θ over a scan time of 30 min.

### 2.5. Catalytic Test

#### 2.5.1. Nitrophenol Calibration Test

UV-visible spectroscopic analyses were performed in a 1 cm path length cuvette using an in-situ Agilent Cary 3500 UV-Vis Spectrometer. The extinction coefficient was determined by using five solutions of different 4-NP concentrations (1.5 × 10^−4^ M, 1.0 × 10^−4^ M, 5.0 × 10^−5^ M, 2.5 × 10^−5^ M, 1.0 × 10^−5^ M), and 4.5∙10^−3^ M in NaBH_4_. The calibration curve was plotted and gave an extinction coefficient of 18,900 M^−1^ cm^−1^, which is close to the literature value of 18,000 M^−1^cm^−1^ [47,48]. The calibration was repeated three times and the average ε was obtained.

#### 2.5.2. Catalytic Reduction of 4-Nitrophenol (Nitrophenols)

An aqueous 4-nitrophenol concentrated solution (0.01 M) was prepared by dissolving 0.1391 g of 4-nitrophenol in distilled water, in a 100 mL volumetric flask. This solution was used to prepare the fresh 4-NP reaction solutions: 500 µL of the initial 4-NP concentrated aqueous solution was withdrawn with a micropipette and diluted to a concentration of 2.0 × 10^−4^ M in a 25 mL volumetric flask. For the preparation of a fresh aqueous solution of NaBH_4_, a 25 mL volumetric flask was used, 8.5 mg of NaBH_4_ was dissolved in deionized water to obtain a solution of 9.0 × 10^−3^ M (4-NP:NaBH_4_ of 1:45 mol/mol). A reference solution for the UV-Vis measurements was prepared by dissolving 4.3 mg of NaBH_4_ in a 25 mL volumetric flask. The following experimental protocol was used: the catalyst (4 mg) was first added inside the beaker, then the 4-NP solution was added. Immediately after, 25 mL of NaBH_4_ solution (9.0 × 10^−3^ M) were added, and this time was considered as the initiation of the reaction. After mixing, the aliquot was withdrawn from the beaker and poured in a quartz cuvette, then subsequently inserted in the UV-Vis spectrometer’s stage. After exactly 2 min and 30 s after the NaBH_4_ addition, the measurement method was started. The optical absorbance of the reaction was set to be automatically measured every 2.5 min for 25 cycles to measure the decrease of 4-nitrophenol concentration as a function of reaction time.

## 3. Results and Discussion

### 3.1. Polymer Synthesis

In this work, polymeric stabilizers based on PVA of different molecular weights were synthesized via solution radical polymerization of vinyl acetate, using different solvents in order to control the molecular weights of the polymers. In this way, during the chain growth, the solvent acts as a chain transfer agent. Table S1 shows the M_n_ of the samples obtained in ethanol, ethyl acetate, and t-butanol. In the polymerization of vinyl acetate in ethyl alcohol, the growing chain can easily abstract a hydrogen from a molecule of the solvent, which leads to a low molecular weight product. The polymer formation in ethyl acetate shows intermediate values of M_n_, due to the similarity between the solvent radicals and vinyl acetate radicals in terms of reactivity and polarity [45]. In the synthesis of polyvinyl-acetate in the presence of t-butanol, the high molecular weight obtained may be due to the high steric hindrance of the hydroxylic group at the tertiary carbon atom. Different mixtures of the above solvents were used (Table 1) in order to modulate the PVAc molecular weight.

The polymer at different percentages of hydrolysis (Table 2) was obtained by direct saponification reaction of PVAc1 modifying the amount of hydroxide added. The synthesized samples were named depending on the hydrolysis degree as PVA-20, PVA-40, PVA-50, PVA-60 (for example, PVA-20 refers to the sample with 20% of the hydroxyl group on the chain). All prepared polymeric stabilizers were subjected to spectroscopic analysis (Figures S1–S3).

### 3.2. Nanoparticles Characterization

#### 3.2.1. Effect of Molecular Weight

UV-visible spectroscopy was used to study the size of gold nanoparticles by the shift of surface plasmon resonance peak (SPR). It was observed that nanoparticles synthesized using PVA with the same hydrolysis degree (99%), but different M_n_, showed a red-shift of SPR when the length of the polymer increases (Figure S4). The characterization of a gold colloidal solution by DLS did not allow evaluating the real diameter of the particles due to the hydrophilicity of the PVA, which promotes the formation of a solvent shell. Therefore, by the DLS analysis, only the hydrodynamic volume of the system could be observed. X-ray diffraction (XRD) confirmed this trend. The main diffraction peak was presented at 38.2° (2θ), which is assigned to the (111) plane of gold. The catalysts synthesized using the low molecular weight PVA did not exhibit a distinct diffraction peak of Au, which can be related to the small crystallite size of AuNPs (Figure S5). Examination of the supported catalysts by transmission electron microscopy (TEM) evidenced a direct correlation between the molecular weights of the polymer and the average diameter of nanoparticles (Figure S6). The characterization of Au/AC catalysts by XPS analysis showed that all Au nanoparticles present on the surface are in the metallic state (Au^0^), as inferred from the Au 4f_7/2_ at 84.0 eV and the related Au 4f_5/2_ at 87.7 eV (Figure S7) [49]. In Table 3 and Table 4, the atomic percentages of gold on the catalyst surface, poly-dispersity index (PI), average crystallite, and particle size of Au are reported, showing the sample labeled as PVA7 (Mn: 92,300) the highest value of exposure gold on the surface.

#### 3.2.2. Effect of Hydrolysis Degree

**Figure 1:**
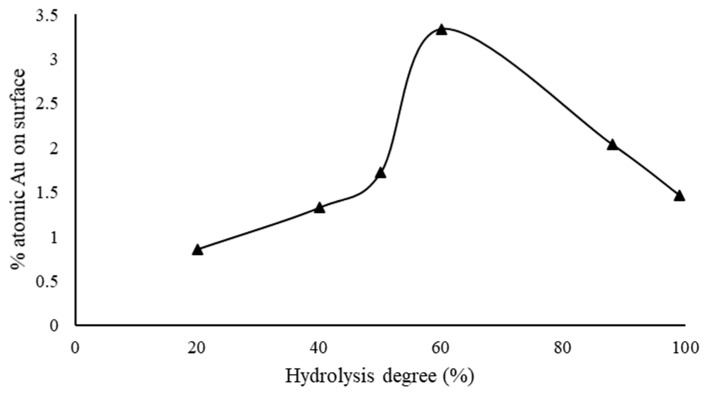
Percentage of atomic Au on the surface versus polyvinyl alcohol hydrolysis degree.

A series of 1% wt. Au/AC catalysts were synthesized using PVAc with the same molecular weight (M_n_ = 23,300), but varying the hydrolysis degree, in order to elucidate the influence of this parameter on the nanoparticle size, morphology, and dispersion on the support. PVA2 and PVA-88 products were used to complete the study since they are commercially available at the same molecular weight. Moreover, PVA2 was called PVA-99 in order to homogenize the name inside the series. Spectroscopic studies performed by UV-visible analysis showed a shift of the plasmonic resonance peak toward the red region of the spectra, in agreement with the decrease in the hydrolysis degree. The reason for this behavior can be due to the smaller number of hydroxyl groups, which allowed the growth of the nanoparticles. In fact, the valuation of the plasmonic shifting described an increase in the Au nanoparticle size (Figure S8). Hydrodynamic volume, detected by DLS, confirmed the trend, but two different competitive effects can be observed: the first one is due to a decrease in the number of functional groups that can interact with the metal surface. The second effect is produced by the formation of solvated shells around the nanoparticles that causes an increase in hydrodynamic volume. Moreover, inter- and intra-molecular hydrophobic interactions of the vinyl acetate sequences can influence the DLS analysis [50,51,52]. The stability of colloidal solutions by zeta potential measurements was studied. A direct proportionality between zeta potential and hydrolysis degree was observed, and the catalyst stabilized using full hydrolyzed PVA resulted in the most stable system due to the great number of hydroxyl groups (Figure S9). Only the XRD analysis performed on the PVA-20 evidenced a distinct diffraction peak for Au, whereas the other samples exhibited a broad peak due to the smaller nanoparticles (Figure S10). TEM images showed an increase in the average nanoparticle size of Au when the percentage of hydroxyl moieties decreases (Figure S11). All XPS spectra, as expected, showed the presence of Au nanoparticles in the metallic state (Au^0^). The peaks are composed of spin-orbit doublets (Au 4f_7/2_, and Au 4f_5/2_). They have characteristic binding energy peaks at 83.8 and 87.5 eV for Au 4f_7/2_ and 4f_5/2_ electrons, respectively (Figure S12) [49]. The volcano plot was observed by correlating the percentage of atomic Au on the surface with the hydrolysis degree of the polymer. The maximum of the curve was attained when the supported nanoparticles were synthesized using PVA-60 (60% hydrolysis degree, Figure 1, Table 5 and Table 6). The hydrolysis degree can directly influence the amount of active phase exposure on the catalyst and therefore the catalytic activity in terms of the number of active sites present.

### 3.3. Catalytic Activity

#### 3.3.1. Effect of Molecular Weight

**Figure 2:**
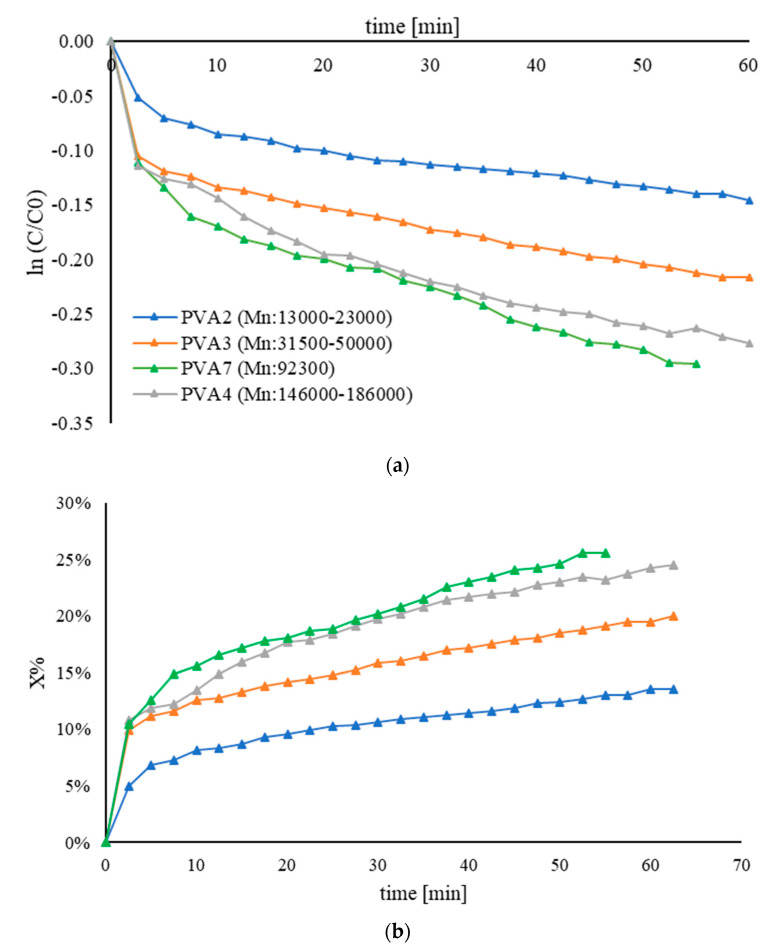
(**a**) Pseudo–first–order kinetic plot and (**b**) conversion plot for the comparison of the activity of Au/AC synthesized using PVA with different molecular weights. The reduction of 4–NP was carried out using 25 mL of 4–NP (2×10^−4^ M), 25 mL of NaBH_4_ (9.0 × 10^−3^ M), an Au/PVA weight ratio of 1:0.65, 4 mg of catalyst, at 25 °C.

Figure 2 displays the catalytic performance of 1 wt.% Au/AC synthesized using PVA fully hydrolyzed with different molecular weights (Table 7). Based on the results reported in Table 3, the sample PVA7 had the highest percentage of exposed gold and therefore the highest k_app_. When the ligand has a higher molecular weight, like PVA4 (Mn: 146,000–186,000), the particle size is bigger, and consequently, the percentage of exposed Au surface is lower, as well as the k_app_. Although an increase in the average nanoparticles size of Au and a different percentage of exposed gold could suggest a great variation of their catalytic performance, it is evident that all catalysts showed a low activity. This trend was probably due to the site-blocking effect produced by the great amount of the hydroxyls group, which minimizes the diffusion of 4-nitrophenolate on the nanoparticle surface.

**Figure 3:**
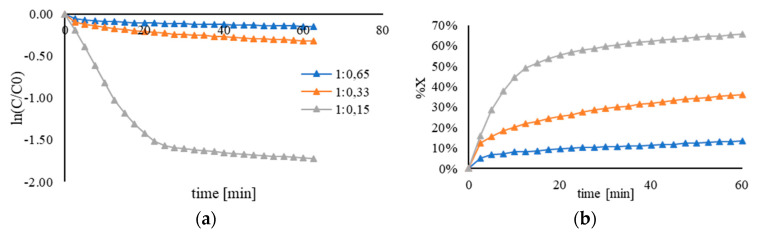
(**a**) Kinetic plot for pseudo–first–order reaction and (**b**) conversion plot using Au/AC synthesized using full hydrolyzed PVA2 (Mw = 13,000–23,000) by varying the Au/PVA weight ratio. Reaction carried out by using 25 mL of 4-NP (2 × 10^−4^ M) and 25 mL of NaBH_4_ (9 × 10^−3^ M), at 25 °C and different PVA amounts: 0.65 mL (Au/PVA (*w*/*w*) = 1:0.65), 0.33 mL (Au/PVA (*w*/*w*) = 1:0.33), 0.15 mL (Au/PVA (*w*/*w*)= 1:0.15).

The effect of the amount of PVA, by varying the Au/PVA weight ratio in the range from 0.15 to 0.65, was investigated in order to explain why these catalysts had a low activity. The Au/PVA weight ratio, and therefore the amount of the polymer on the catalyst surface, could influence the catalytic performance in terms of the availability of active sites. The sample PVA2 (Mn = 13,000–23,000) was chosen based on the small average nanoparticle size of Au to investigate the effect of this parameter. The catalytic performance was enhanced when the Au/PVA weight ratio was decreased. The supported Au nanoparticles obtained with an Au/PVA weight ratio of 1:0.15 presented an apparent kinetic constant of 5.3 × 10^−2^ min^−1^. This value was more than ten times higher than the catalyst prepared with 1:0.65 (1.8∙× 10^−4^ min^−1^) and 1:0.33 (3.9∙10^−4^ min^−1^) weight ratio of Au/PVA (Figure 3, Table 8). The increase in the apparent rate constant observed with respect to the rest of Au-based catalysts allows us to confirm the significant contribution of polymeric stabilizers in terms of affecting the availability of Au active sites, which can be explained by the potential coverage of the active sites by the stabilizer. The polymer around the nanoparticles, due to the hydrophilic groups, can produce electronic and steric effects, which can inhibit the catalytic activity.

The catalysts synthesized using PVA2 (Mn = 13,000–23,000) with different Au:PVA weight ratios were characterized in order to explain the observed catalytic performance. DLS analysis showed an increase in the hydrodynamic volume, due to the decrease of the polymer amount around the nanoparticles. Moreover, the increase in the size is due to the smaller efficiency of PVA as a stabilizer which is influenced by the smaller amount of PVA present in the solution. From TEM analysis, an increase in nanoparticles size was observed for the sample prepared with a low amount of polymer (Figure S13). Moreover, a broad diffraction peak was observed in the X-ray diffraction pattern due to the small dimensions of nanoparticles (Figure S14). XPS spectra showed that there was only metallic Au on the catalyst surface, with values in the range of 84.1 to 84.2 (Figure S15). The atomic percentage of gold on the surface was similar (1.46 and 1.47) for the samples prepared using an Au:PVA weight ratio of 1:0.15 and 1:0.65. The catalyst synthesized with a ratio of 1:0.33 had the highest percentage of Au (3.35) (Table 9). However, it showed a lower catalytic activity than the sample with an Au:PVA of 1:0.15. The variation of polymer amount had a significant impact in terms of particle size and the exposed surface of Au, with an increase in particle size and a decrease in exposed Au surface when the amount of polymer decreased significantly. From these results, it seems that the amount of polymer could influence the diffusion of reactants to the particle surface. The reagent, in this case, 4-NP, to diffuse from the reaction medium to the gold surface had to cross the polymer layer around the Au nanoparticle. A low PVA amount could promote this migration and therefore influence the catalytic activity.

#### 3.3.2. Effect of Hydrolysis Degree

**Figure 4:**
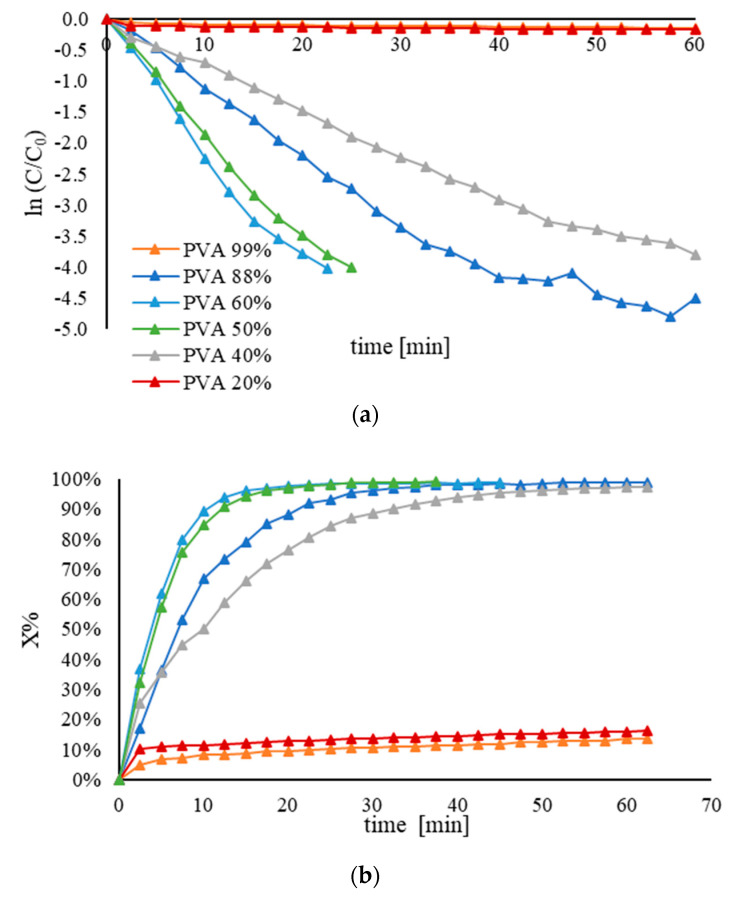
(**a**) Pseudo–first−order kinetic plot and (**b**) conversion plot for the comparison of the activity of Au/AC synthesized using PVA with different hydrolysis degrees. The reduction of 4–NP was carried out using 25 mL of 4–NP (2 × 10^–4^ M), 25 mL of NaBH_4_ (9.0 × 10^−3^ M), an Au/PVA weight ratio of 1:0.65, 4 mg of catalyst, at 25 °C.

**Figure 5:**
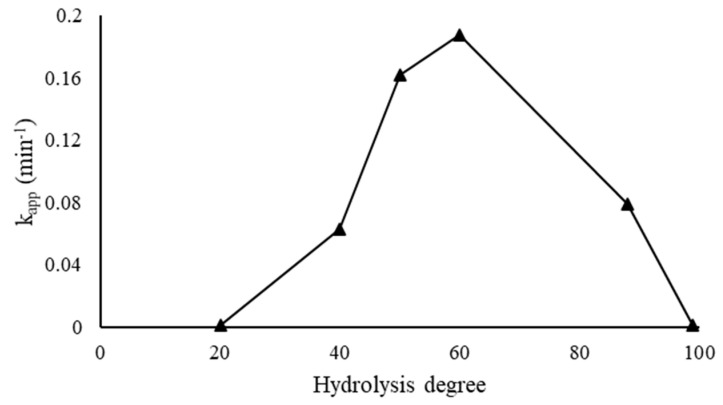
Volcano plot for the effect of PVA hydrolysis degree on the catalytic activity, reaction carried out using 25 mL of 4–NP (2 × 10^−4^ M), 25 mL of NaBH_4_ (9.0 × 10^−3^ M), an Au/PVA weight ratio of 1:0.65, 4 mg of catalyst, at 25° C.

**Figure 6:**
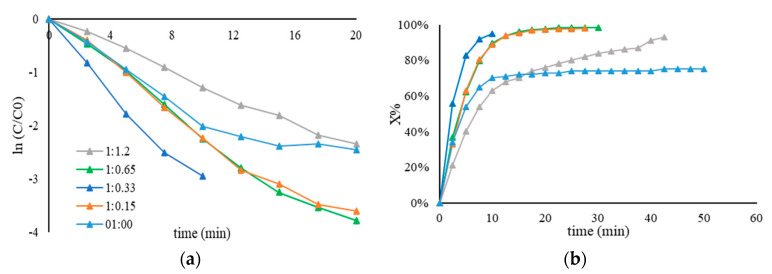
(**a**) Pseudo–first−order kinetic plot and (**b**) conversion plot using Au/AC synthesized using PVA-60 (derived from a PVAc Mn = 23,300) by varying the Au/PVA weight ratio. Reaction carried out by using 25 mL of 4–NP (2 × 10^−4^ M) and 25 mL of NaBH_4_ (9 × 10^−3^ M), 25 °C and different PVA amounts: 1.2 mL (Au/PVA (*w*/*w*)= 1:1.2), 0.65 mL (Au/PVA (*w*/*w*)= 1:0.65), 0.33 mL (Au/PVA (*w*/*w*)= 1:0.33), 0.15 mL (Au/PVA (*w*/*w*)= 1:0.15), and 0 mL (Au/PVA (*w*/*w*)= 1:0).

A series of 1% wt. Au/AC catalysts were synthesized by varying the hydrolysis degree of PVAc1 (M_n_ = 23,300) in the range of 20% to 99%, in order to evaluate its influence on the catalytic activity (Table 10). Figure 4 shows that the percentages of hydrolysis modified the catalytic performance. In particular, a volcano plot was observed, and the maximum of the curve was achieved for a hydrolysis degree of 60% (Figure 5). The same trend was spotted correlating the atomic percentage of Au on the catalyst surface with the hydrolysis degree of the PVA, and, in this case, the maximum also corresponded to the sample synthesized with PVA-60. Analyzing the curve between 20% and 60% of hydrolysis degree, k_app_ increased as the mean particle size of Au was decreased from 9.6 to 3.9. The decrease in nanoparticle size and the decrease in the amount of exposed gold may explain this trend. Upon further increasing the hydrolysis degree from 60% until fully hydrolyzed PVA (99%), the rate of the reaction decreased due to the formation of the bond between the solvent molecules and the hydroxyl groups, which inhibit the catalytic activity. Based on the catalytic studies after optimization, the Au/AC sample with PVA-60 as the desired hydrolysis degree was chosen to conduct the following studies. Subsequently, the Au/PVA-60 weight ratio was further studied and optimized in order to choose the optimal amount of polymeric stabilizer. The Au/PVA-60 weight ratio was varied from 1:0 to 1:1.2 while maintaining the other experimental parameters. As depicted in Figure 6, it was found that when the Au/PVA-60 weight ratio was decreased from 1:0.65 to 1:0.33 the k_app_ was increased from 0.2 to 0.3 min^−1^. The other values were lower than the k_app_ values observed at the Au/PVA-60 weight ratio of 1:0.65 (Table 11). The sample synthesized using PVA-60 with a 1:0.33 weight ratio was considered the best catalyst and it was used to investigate the substituent effect on its catalytic performance. The samples were further characterized in order to establish a structure-activity relationship and, therefore, to explain the catalytic activity observed in the samples prepared using PVA-60 with different Au:PVA weight ratios. The decrease in the PVA-60 amount corresponds to the increase in hydrodynamic volume detected by DLS (Table 11). Moreover, the Au:PVA weight ratio can influence zeta potential and furthermore the stability of the catalysts. In Figure S16, a direct proportionality was observed varying the Au:PVA weight ratio of the catalysts. The sample synthesized without the polymeric stabilizer showed a low stability due to the absence of steric hindrance derived from the macromolecules. An increase in nanoparticle size was observed by TEM images when the weight ratio Au:PVA decreased (Figure S17). The same trend was observed in XRD patterns. The samples prepared with the ratios of 1:1.2 and 1.0.65 showed a broad peak, whereas a well-defined peak was observed for a lower Au:PVA ratio (Figure S18). Only metallic gold was presented in XPS spectra (Figure S19). A variation of surface Au coverage was observed which depends on the amount of the stabilizer used and the particle size of Au.

The catalyst obtained using an Au:PVA weight ratio of 1:0.33 showed the best catalytic performance and had a low value of surface exposure of Au (Table 12). It is evident that the PVA amount used during the nanoparticle preparation influences the resulting particle size of the supported gold nanoparticles, the gold surface coverage, and indirectly the catalytic mechanism in terms of diffusion of the reagents on the catalyst surface. From these studies, the optimum particle size is in the range of 5 nm with a low Au:PVA weight ratio (1:0.33).

#### 3.3.3. Substituent Effect

**Figure 7:**
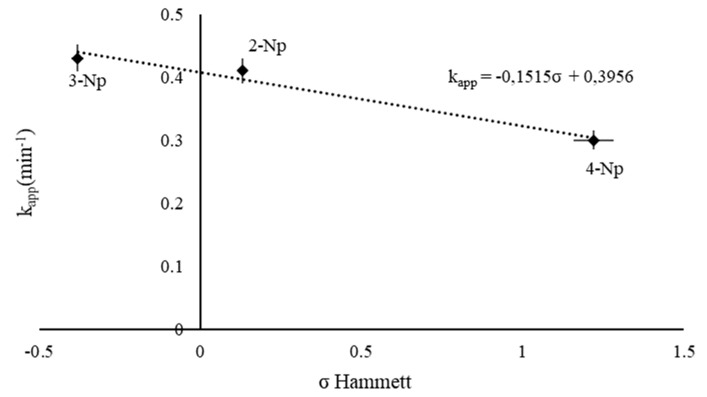
Hammett plot for the effect of the substituent in nitrophenol’s isomer on the catalytic activity, reaction carried out using 25 mL of NP (2 × 10^−4^ M), 25 mL of NaBH_4_ (9.0 × 10^−3^ M), Au/PVA weight ratio of 1:0.33, 4 mg of catalyst, at 25 °C.

To analyze the substituent effect on the catalytic activity of 1% wt. Au/AC, with an Au:PVA-60 weight ratio of 1:0.33, different isomers of nitrophenol were investigated. Furthermore, the Hammett equation was used for the elucidation of the catalytic mechanism. The different conversion rate of each nitrophenol isomer depends on the stability of the corresponding nitrophenolate ion. For 2- and 4-nitrophenolates, the negative charge on the phenoxide ion can be delocalized into the nitro group, and therefore stabilized due to the resonance effect. In addition, a stronger inductive effect is present in 2-nitrophenolate due to the shorter distance between the substituent group and the reactive center. The stronger inductive effect makes the nitrogen atom more positively charged, and therefore more reactive. In the case of 3-nitrophenolate, there is no resonance stabilization of the negative charge into the nitro group, and only a small inductive effect can be observed. For this reason, the rate of hydrogenation for the nitrophenol isomers follows the order *3- > 2- > 4-* [53,54] (Table 13). The Hammett plot presented in Figure 7 shows a good correlation between the revised σ values and the rate constant, with a negative slope: ρ = −0.15. These results suggest that a positive charge develops in the transition state, or a negative charge is lost.

### 3.4. Reusability Tests

**Figure 8:**
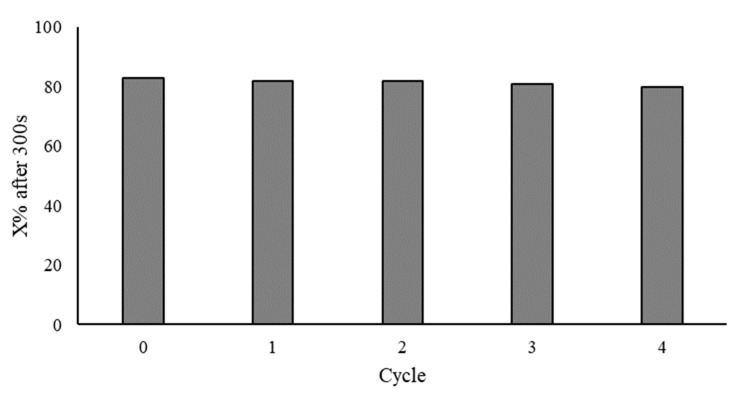
Plot of conversion per each run of the reusability tests.

To complete the investigation on the Au/AC catalyst synthesized using PVA-60 with a 1:0.33 Au/PVA weight ratio, a reusability study was also carried out. These tests were conducted in a round bottom flask in order to increase the amount of solution and catalyst used. The conversion reached, after a defined reaction time of 300 s, was used to compare the catalytic activity of the five recycling tests. As displayed in Figure 8, the conversion remained constant, at around 80%. Moreover, Microwave Plasma Atomic Emission Spectroscopy (MP-AES) and XPS analyses were performed for the reaction solution and the used catalyst, respectively. MP-AES revealed that only traces of Au (below 10 ppb) were detected in the reaction filtrate. This result is in agreement with the stable catalytic activity showed during the reusability tests. XPS analysis of the used catalyst showed no variation to the spectrum of the fresh catalyst. The amount of gold changed from 1.38 to 1.87, the polymer was probably removed, and, therefore, increased the percentage of Au on the surface (Figure S20; Table S2). Moreover, by TEM images, nanoparticle sintering was observed, since the size was changed from 5.2 to 7.8 nm after the fifth cycle (Figure S21).

## 4. Conclusions

The results obtained in this study demonstrate that polymeric stabilizers influence the nanoparticle size and activity of supported gold nanoparticles. Several characterization techniques were used to characterize AuNPs synthesized using PVA with a low molecular weight and high hydrolysis degree. Although the presence of a high amount of hydroxyl groups enhanced the stability of the gold colloidal solution, the catalytic activity was inhibited due to the steric and electronic effects of the polymer chains. The study also shows that a volcano plot was obtained by correlating the PVA hydrolysis degree with apparent kinetic constant relative to 4-nitrophenol reduction. The maximum was identified in the sample synthesized with PVA hydrolyzed at 60% (M_n_= 23,300). The Au:PVA weight ratio was studied in order to evaluate how the amount of the polymer can influence the availability of active sites. The sample with a 1:0.33 ratio resulted in the best catalyst in terms of activity. The effect of substituents on the aromatic ring was investigated and the 3-nitrophenol was the most reactive substrate due to the absence of resonance and inductive effect. Reusability tests were conducted on the PVA-60 with an Au:PVA ratio of 1:0.33 and a little variation was observed in activity after five catalytic cycles. The synthesized catalyst result was very efficient and thereby showed a high potential for a variety of catalytic and environmental applications. In addition, these findings emphasize the importance of ligand−nanoparticle interrelationships in the catalytic mechanism of supported nanoparticles.

## Figures and Tables

**Table 1:** Solvent mixtures used in the polyvinyl-acetate (PVAc) synthesis in order to control the molecular weight.

	*PVAc1*	*PVAc2*	*PVAc3*	*PVAc4*
**Ethanol**	100	75	-	-
**Ethyl Acetate**	-	25	50	-
***t*-Butanol**	-	-	50	100

**Table 2:** Molecular characteristics of polyvinyl alcohol (PVA) used as polymeric stabilizers. * indicates a commercial product, ** indicates the molecular weight of starting material.

Sample	$\bar{M_{n}}$	Hydrolysis Degree
PVA2 *	13,000–23,000	99
PVA3 *	31,000–50,000	99
*PVA7*	92,300 **	99
PVA4 *	146,000–186,000	99
PVA-88 *	13,000–23,000	88
*PVA-60*	23,300 **	60
*PVA-50*	23,300 **	50
*PVA-40*	23,300 **	40
*PVA-20*	23,300 **	20

**Table 3:** X-ray photoelectron spectroscopy (XPS) data for 1% wt. Au/activated carbon (AC) synthesized using PVA-99 with different molecular weights.

	$\bar{M_{n}}$	BE Au 4f_7/2_ (eV)	Au on Surface (% Atomic)	C on Surface (% Atomic)	Surface Atomic Ratio Au/C
**PVA2**	13,000–23,000	84.1	1.47	91.2	0.02
**PVA3**	31,000–50,000	84.1	1.22	91.4	0.02
**PVA7**	92,300	84.0	2.31	89.5	0.03
**PVA4**	146,000–186,000	84.1	1.13	92.3	0.01

**Table 4:** Comparison between poly-dispersity index (PI), dynamic light scattering (DLS), X-ray diffraction (XRD), and transmission electron microscopy (TEM) diameter of gold nanoparticles synthesized using PVA with different molecular weights.

	$\bar{M_{n}}$	PI_DLS_	d_DLS_ (nm)	d_TEM_ (nm)	d_XRD_ (nm)
**PVA2**	13,000–23,000	0.34	6.0	3.2	-
**PVA3**	31,000–50,000	0.83	3.9	3.8	-
**PVA7**	92,300	0.59	6.3	4.2	4.4
**PVA4**	146,000–186,000	0.46	7.5	5.7	5.9

**Table 5:** X-ray photoelectron spectroscopy (XPS) data for 1% wt. Au/AC synthesized using PVA-2 (M_n_ = 13,000–23,000) with different hydrolysis degrees.

	Hydrolysis Degree	BE Au 4f_7/2_ (eV)	Au on Surface (% Atomic)	C on Surface (% Atomic)	Surface Atomic ratio Au/C
PVA-99	99	84.1	1.47	91.2	0.02
PVA-88	88	84.1	2.04	89.7	0.02
PVA-60	60	84.1	3.34	90.2	0.04
PVA-50	50	84.1	1.72	92.2	0.02
PVA-40	40	84.0	1.33	90.1	0.02
PVA-20	20	84.0	0.86	94.1	0.01

**Table 6:** Comparison between poly-dispersity index (PI), DLS, XRD, and TEM diameter of gold nanoparticles obtained using PVA with different hydrolysis degrees.

	Hydrolysis Degree	PI_DLS_	d_DLS_ (nm)	d_TEM_ (nm)	d_XRD_ (nm)
PVA-99	99	0.34	6.0	3.2	-
PVA-88	88	0.68	5.1	3.4	-
PVA-60	60	0.30	5.1	3.9	-
PVA-50	50	0.47	9.4	4.2	-
PVA-40	40	0.59	12.1	4.3	-
PVA-20	20	0.51	13.5	9.6	9.6

**Table 7:** Values of the apparent constant and conversion for pseudo-first-order reactions using PVA with different molecular weights.

	$\bar{M_{n}}$	d_TEM_ (nm)	Au on Surface (% Atomic)	k_app_ (min^−1^)	X%
**PVA2**	13,000–23,000	3.2	1.47	1.8 × 10^−3^ ± 4 × 10^−4^	14 ± 1
**PVA3**	31,000–50,000	3.8	1.22	3.1 × 10^−3^ ± 8 × 10^−4^	24 ± 1
**PVA7**	92,300	4.2	2.31	3.7 × 10^−3^ ± 4 × 10^−4^	22 ± 1
**PVA4**	146,000–186,000	5.7	1.13	2.4 × 10^−3^ ± 3 × 10^−4^	20 ± 1

**Table 8:** Values of the apparent constant and conversion for pseudo-first-order reactions using full hydrolyzed PVA2 (Mw = 13,000–23,000) by varying the Au/PVA weight ratio.

	$\bar{M_{n}}$	Au:PVA	PI_DLS_	d_DLS_ (nm)	d_TEM_ (nm)	d_XRD_ (nm)	Au on Surface (% Atomic)	k_app_ (min^−1^)	X%
**PVA2**	13,000–23,000	1:0.65	0.34	6.0	3.2	-	1.47	1.8∙10^−3^ ± 4∙10^−4^	14 ± 1
**PVA2**	13,000–23,000	1:0.33	0.36	5.6	3.8	4.0	3.35	3.9∙10^−3^ ± 2∙10^4^	37 ± 3
**PVA2**	13,000–23,000	1:0.15	0.78	4.2	5.0	5.6	1.46	5.3∙10^−2^ ± 2∙10^−3^	66 ± 6

**Table 9:** XPS data for 1% wt. Au/AC synthesized using PVA2 (Mn = 13,000–23,000) with different Au:PVA weight ratios.

	Au:PVA (*w*/*w*)	BE Au 4f_7/2_ (eV)	Au on Surface (% Atomic)	C on Surface (% Atomic)	Surface Atomic Ratio Au/C
**PVA2**	1:0.65	84.1	1.47	91.2	0.02
**PVA2**	1:0.33	84.2	3.35	89.9	0.04
**PVA2**	1.0.15	84.1	1.46	93.2	0.02

**Table 10:** Values of the apparent kinetic constant and conversion for pseudo-first-order reactions using PVA with different hydrolysis degrees.

	Hydrolysis Degree	d_TEM_ (nm)	Au on Surface (% atomic)	k_app_ (min^−1^)	X%
**PVA-99**	99	3.2	1.47	1.8∙10^−3^ ± 4∙10^−4^	14 ± 1
**PVA-88**	88	3.4	2.04	7.9∙10^−2^ ± 1∙10^−3^	97 ± 1
**PVA-60**	60	3.9	3.34	0.20 ± 4∙10^−3^	99 ±0.4
**PVA-50**	50	4.2	1.72	0.10 ± 4∙10^−2^	99 ± 1
**PVA-40**	40	4.3	1.33	6.3∙10^−2^ ± 9∙10^−3^	97 ± 1
**PVA-20**	20	9.6	0.86	1.4∙10^−3^ ± 2∙10^−4^	16 ± 0.2

**Table 11:** Values of the apparent constant and conversion for pseudo-first-order reactions using PVA-60 (derived from a PVac Mn = 23,300), by varying the Au/PVA weight ratio.

	Hydrolysis Degree	Au:PVA	PI_DLS_	d_DLS_ (nm)	d_TEM_ (nm)	d_XRD_(nm)	Au on Surface (% Atomic)	k_app_ (min^−1^)	X%
**PVA60**	60	1:1.2	0.52	5.1	3.5	-	1.87	0.12 ± 5∙10^−2^	93 ± 1
**PVA60**	60	1:0.65	0.30	5.1	3.9	-	3.34	0.20 ± 4∙10^−3^	99 ± 0.4
**PVA60**	60	1:0.33	0.78	7.5	5.2	5.9	1.38	0.30 ± 4∙10^−2^	99 ± 0.3
**PVA60**	60	1:0.15	0.46	8.2	6.8	7.3	1.62	0.19 ± 2∙10^−2^	99 ± 1
**PVA60**	60	1:0	0.32	10.7	8.1	8.9	1.06	0.13 ± 4∙10^−2^	75 ± 1

**Table 12:** XPS data for 1% wt. Au/AC synthesized using PVA-60 (derived form a PVAc Mn = 23,300) with different Au:PVA weight ratios.

	Au:PVA (w/w)	BE Au 4f_7/2_ (eV)	Au on Surface (% Atomic)	C on Surface (% Atomic)	Surface Atomic Ratio Au/C
**PVA-60**	1:1.2	84.1	1.87	92.9	0.02
**PVA-60**	1:0.65	84.1	3.34	90.2	0.04
**PVA-60**	1:0.33	84.1	1.38	93.2	0.02
**PVA-60**	1.0.15	84.1	1.62	93.7	0.02
**PVA-60**	1:0	84.1	1.06	94.9	0.01

**Table 13:** Apparent kinetic constant and conversion for pseudo-first-order-reactions using PVA-60 with an Au/PVA weight ratio of 1.0.33.

	σ [54]	k_app_ (min^−1^)	X%
**4-nitrophenol**	1.22	0.30 ± 4∙10^−2^	99 ± 0.3
**2-nitrophenol**	0.13	0.41± 2∙10^−2^	99 ± 0.1
**3-nitrophenol**	−0.38	0.43± 2∙10^−2^	99 ± 0.3

## Data Availability

Data are contained within the article.

## Supplementary Materials

The following are available online at https://www.mdpi.com/article/10.3390/nano11040879/s1, Figure S1: (a) ^1^H-NMR (Proton nuclear magnetic resonance) in CDCl_3_ and (b) FT-IR (Furier transform-infrared spectroscopy) spectra of polyvinyl acetate (PVAc); Table S1: GPC (Gel Permeation Chromatography) data; Figure S2: ^1^H-NMR spectrum in d_6_-DMSO-D_2_O of full hydrolyzed polyvinyl alcohol; Figure S3: FT-IR spectra of polyvinyl alcohol partially hydrolyzed: focus on the C=O stretching at 1729 cm^−1^; Figure S4: UV-visible spectra and positions of the surface plasmon resonance peak of the Au colloidal nanoparticles obtained by PVA as a function of molecular weight; Figure S5: XRD patterns of the Au supported colloidal nanoparticles obtained by PVA as a function of molecular weight; Figure S6: TEM images and particle size distributions of Au/AC synthesized using different molecular weights: (a) M_n_= 13,000–23,000; (b) M_n_ = 31,000–50,000; (c) M_n_ = 9,2300 and (d) M_n_= 146,000–186,000; Figure S7: XPS spectra for 1% wt. Au/AC synthesized using PVA-99 with different molecular weights; Figure S8: UV-visible spectra relative to gold nanoparticles obtained by PVA (Mn: 23,300) with different hydrolysis degrees: focus on surface plasmon resonance peak; Figure S9: Zeta potential (ζ) of gold nanoparticles versus polyvinyl alcohol hydrolysis degree (Mn: 23,300); Figure S10: XRD patterns of the Au supported nanoparticles obtained by PVA (Mn: 23,300) as a function of the hydrolysis degree; Figure S11: TEM images and particle size distributions of Au/AC synthesized using different hydrolysis degrees: (a) 20%; (b) 40%; (c) 50%; d) 60%; (e) 88%; (f) 99%; Figure S12: XPS spectra for 1% wt. Au/AC synthesized using PVA (Mn: 23,300) with different hydrolysis degrees; Figure S13: TEM images and particle size distributions of Au/AC synthesized using PVA2 (Mn = 13,000–23,000) with different Au:PVA weight ratios: a) ratio 1:0.15 (Au:PVA); b) ratio 1:0.33 (Au:PVA); Figure S14: XRD patterns of the Au supported nanoparticles obtained by PVA2 (Mn: 13,000–23,000) with different Au:PVA weight ratios; Figure S15: XPS spectra for 1% wt Au/AC synthesized using PVA2 (13,000–23,000) with different Au:PVA weight ratios; Figure S16: Zeta potential (ζ) of gold nanoparticles versus amount of PVA-60 (M_n_: 23,300) in Au:PVA weight ratio; Figure S17: TEM images and particle size distributions of Au/AC synthesized using PVA-60 (Mn = 23,300) with different Au:PVA weight ratios: (a) ratio 1:1.2 (Au:PVA); (b) ratio 1:0.33 (Au:PVA); (c) 1:0.15 (Au:PVA); (d) 1:0 (Au:PVA); Figure S18: XRD patterns of the Au supported nanoparticles obtained by PVA-60 (Mn: 23,300) with different Au:PVA weight ratios; Figure S19: XPS spectra for 1% wt. Au/AC synthesized using PVA-60 (23,000) with different Au:PVA weight ratios; Figure S20: XPS spectra for 1% wt. Au/AC synthesized using PVA-60 (23,000) with Au:PVA of 1:0.33: comparison between fresh and used catalyst; Table S2: XPS data for 1% wt. Au/AC synthesized using PVA-60 (Mn = 23,300) with Au:PVA of 1:0.33: comparison between fresh and used catalyst; Figure S21: TEM images and particle size distributions of Au/AC used catalyst synthesized with PVA-60 (Mn = 23,300) Au:PVA of 1:0.33.
