# Supplementary material for: Effect of Polyvinyl Alcohol Ligands on Supported Gold Nano-Catalysts: Morphological and Kinetics Studies
Source: Nanomaterials (Basel). 2021 Mar 30;11(4):879. doi: 10.3390/nano11040879 (PMC8066135; doi:10.3390/nano11040879)
Supplement: Supplementary file 1 [file nanomaterials-11-00879-s001.pdf]

nanomaterials

Supplementary Materials

Effect of Polyvinyl Alcohol Ligands on Supported Gold Nano-Catalysts: Morphological and Kinetics Studies

Stefano Scurti [1], Eleonora Monti [1], Elena Rodríguez-Aguado [2], Daniele Caretti [1,*], Juan Antonio Cecilia [2] and Nikolaos Dimitratos [1,*]

[1] Industrial Chemistry "Toso Montanari" Department, University of Bologna, Viale Risorgimento 4, 40126 Bologna, Italy; stefano.scurti2@unibo.it (S.S.); eleonora.monti8@unibo.it (E.M.)

[2] Departamento de Química Inorgánica, Cristalografía y Mineralogía (Unidad Asociada al ICP-CSIC), Facultad de Ciencias, Universidad de Málaga, Campus de Teatinos, 29071 Málaga, Spain; aguadoelena5@gmail.com (E.R.-A.); jacecilia@uma.es (J.A.C.)

* Correspondence: daniele.caretti@unibo.it (D.C.); nikolaos.dimitratos@unibo.it (N.D.)

-Poly vinyl alcohol synthesis and characterization

To characterize polymer synthesized by radical polymerization of vinyl acetate NMR as well as FT-IR spectra were recorded and reported in **Figure S1**. By ^{1}H-NMR spectrum, reference peak of the CH_3 derived from acetate group was observed at 2 ppm. The other aliphatic hydrogen signals were observed at 1.75 ppm and 4.9 ppm. **Figure S1b** shows FT-IR spectra of PVAc where typical reference bands at 1729 and 1300–1000 cm^{-1} related to the stretching vibrations of C=O and C−O group are present.

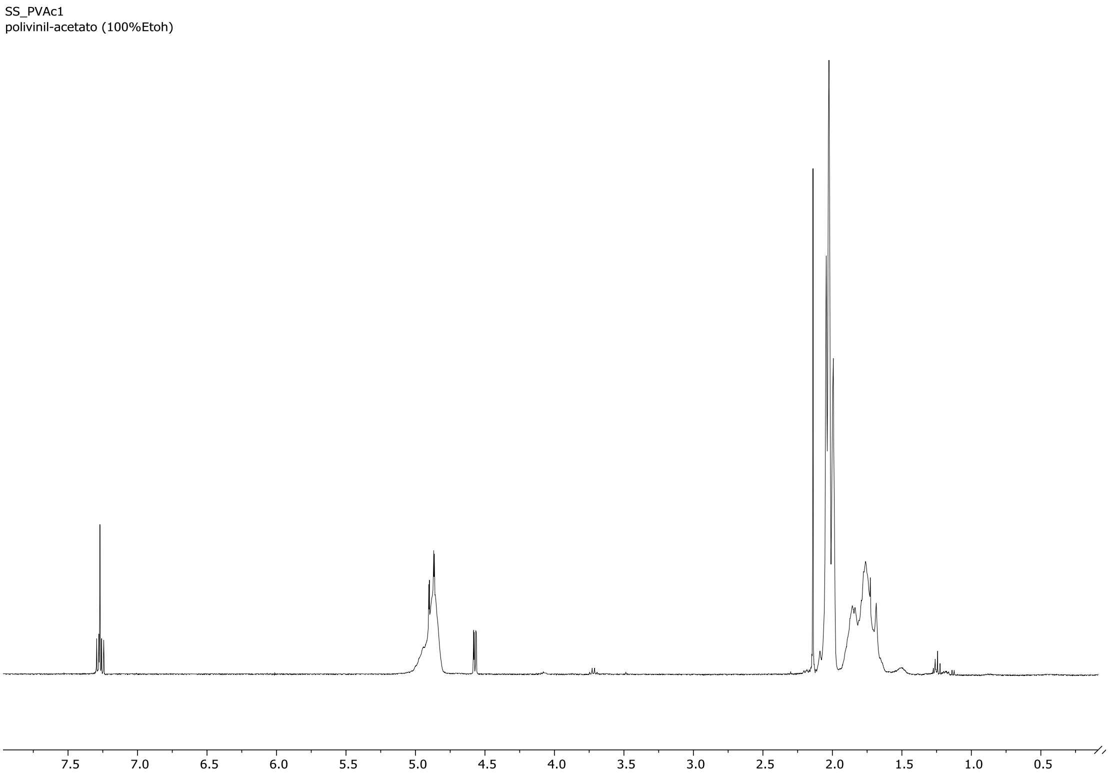

(**a**)

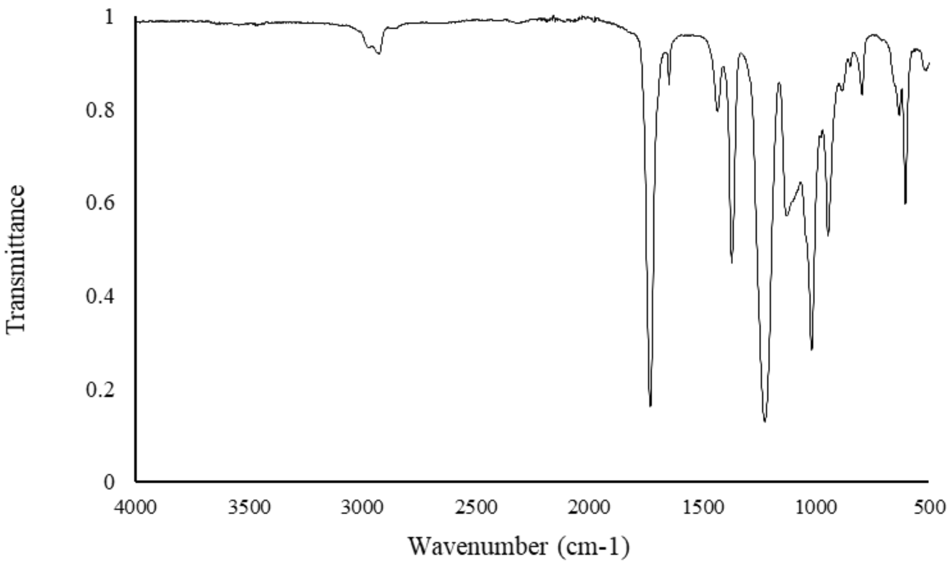

(**b**)

Figure S1. (**a**) ^{1}H-NMR (Proton nuclear magnetic resonance) in $CDCl_3$ and (**b**) FT-IR (Furier Transform Infra-Red Spectroscopy) spectra of poly-vinyl acetate (PVAc).

Table 1. GPC (Gel Permeation Chromatography) data.

	$\overline{M_n}$	$\overline{M_w}$	*Polidispersity*
PVAc1	23,300	44,700	1.9
PVAc2	32,800	60,500	1.8
PVAc3	92,300	221,500	2.4
PVAc4	161,500	616,400	3.8

Poly-vinyl alcohol synthesized by direct saponification process was characterized by means of NMR and FT-IR spectroscopies. By ^{1}H-NMR spectrum reported in **Figure S2.** it is possibile to confirm the presence of full-hydrolyzed PVA due to the absence of CH_3 peak at 2 ppm.

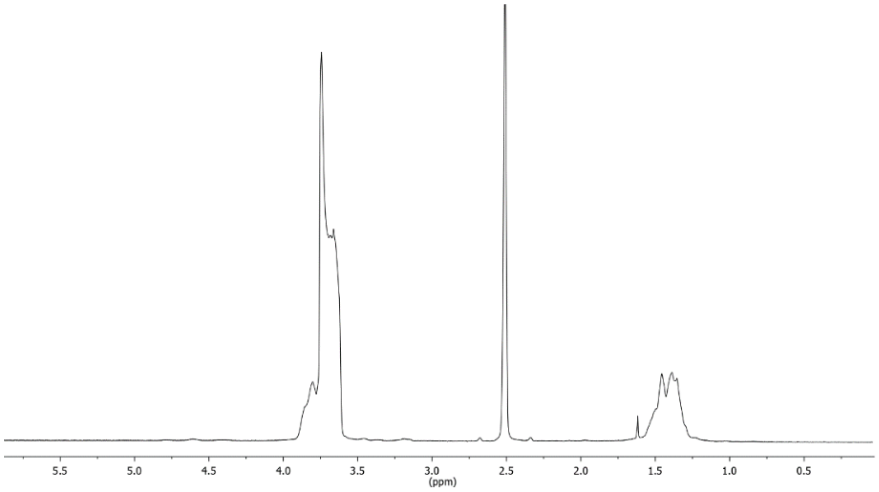

Figure S2. ^{1}H-NMR spectrum in d_6-DMSO-D_2O of full hydrolyzed poly-vinyl alcohol.

By using a reference peak, the intensity of C=O stretching band at 1729 cm^{-1}, the hydrolysis degree in PVA partially hydrolyzed was evaluated. **Figure S3** showed the FT-IR spectra of the synthesized polymers.

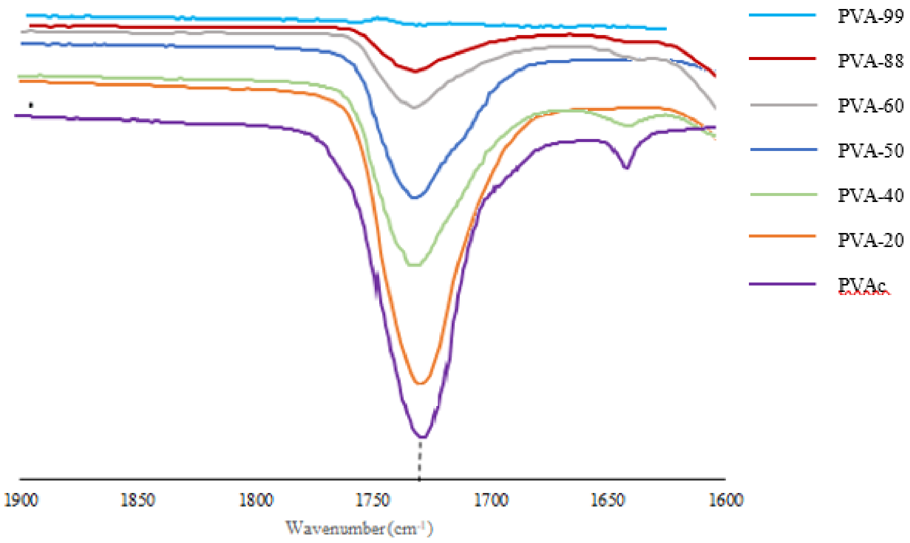

Figure S3. FT-IR spectra of poly-vinyl alcohol partially hydrolysed: focus on the C=O stretching at 1729 cm^{-1}.

-Nanoparticles characterization: effect of molecular weight

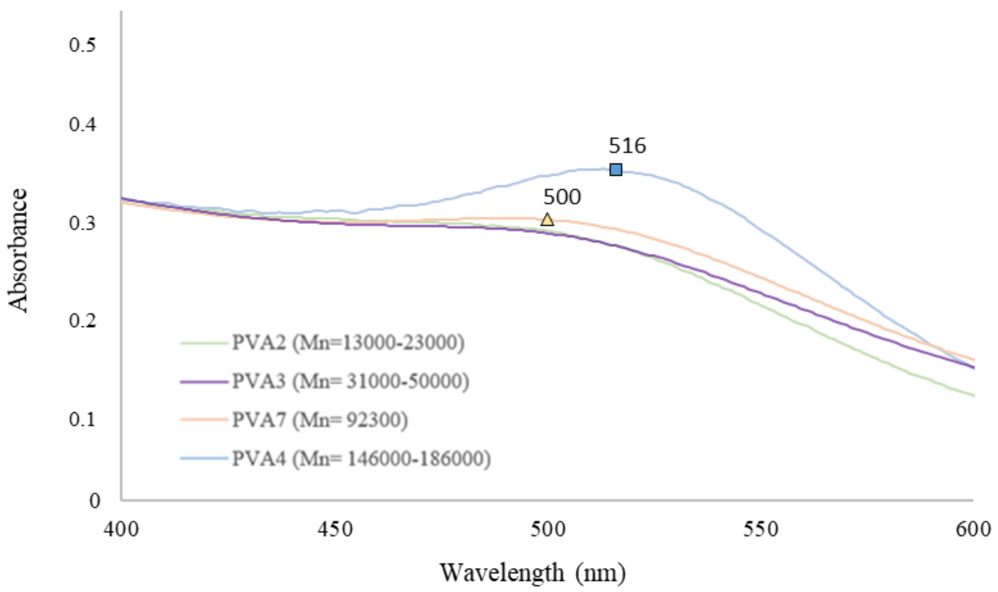

Figure S4. UV-visibile spectra and positions of the surface plasmon resonance peak of the Au colloidal nanoparticles obtained by PVA as a function of molecular weight.

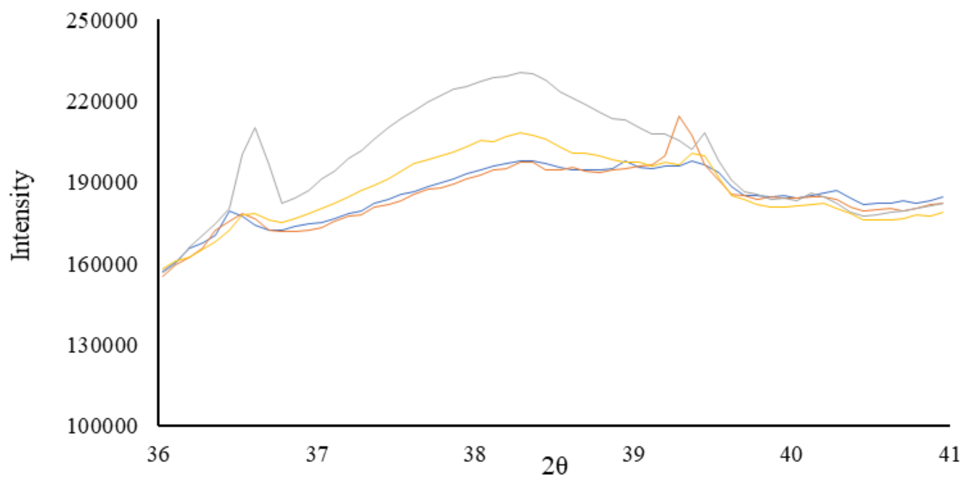

Figure S5. XRD patterns of the Au supported colloidal nanoparticles obteined by PVA as a function of molecular weight.

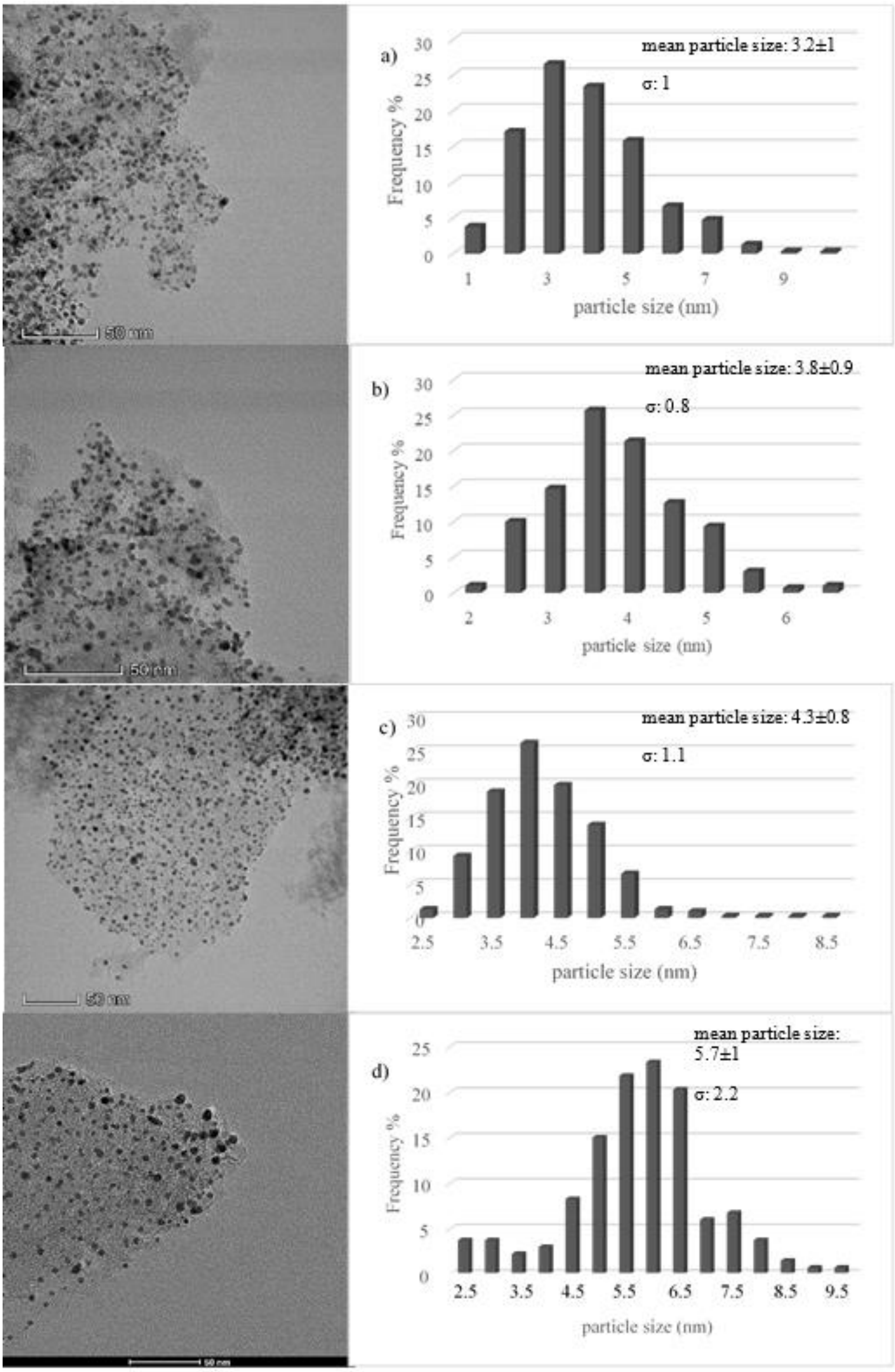

Figure S6. TEM images and particle size distributions of Au/AC synthesized using different molecular weights: (**a**) M_n= 13,000–23,000; (**b**) M_n= 31,000–50,000; (**c**) M_n= 92,300 and (**d**) M_n= 146,000–186,000.

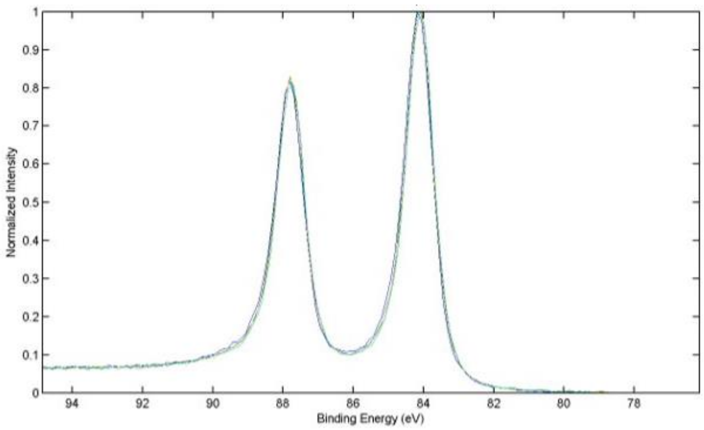

Figure S7. XPS spectra for 1% wt Au/AC synthesized using PVA-99 with different molecular weights.

-Nanoparticles characterization: effect of hydrolysis degree

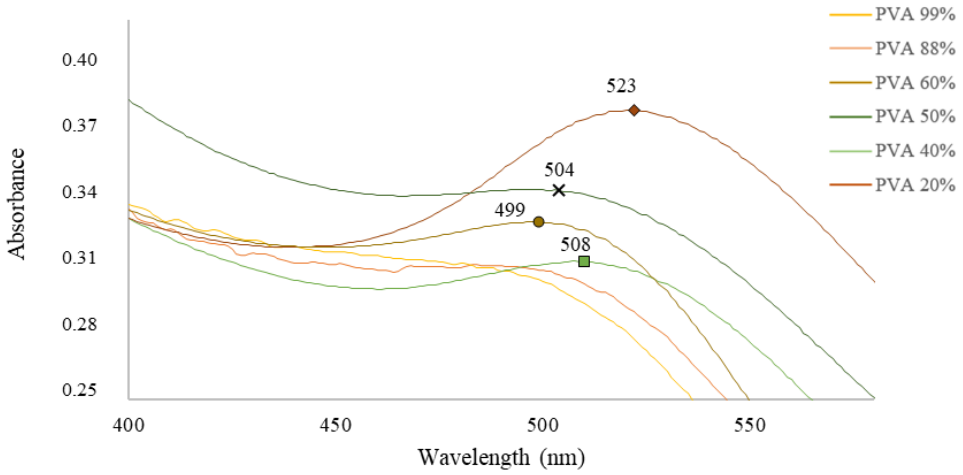

Figure S8. UV-visibile spectra relative to gold nanoparticles obtained by PVA (Mn:23,300) with different hydrolysis degree: focus on surface plasmon resonance peak.

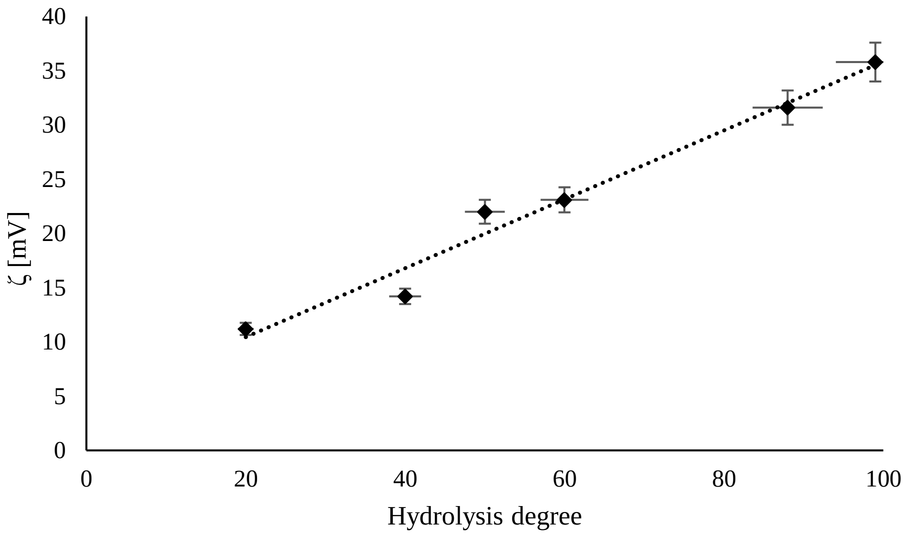

Figure S9. Zeta potential (ζ) of gold nanoparticles versus poly-vinyl alcohol hydrolysis degree (Mn:23,300).

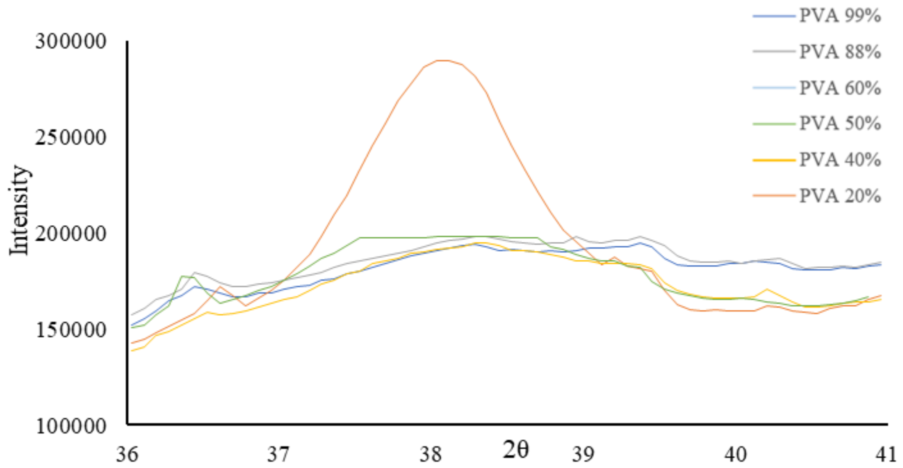

Figure S10. XRD patterns of the Au supported nanoparticles obtained by PVA (Mn:23,300) as a fucntion of the hydrolysis degree.

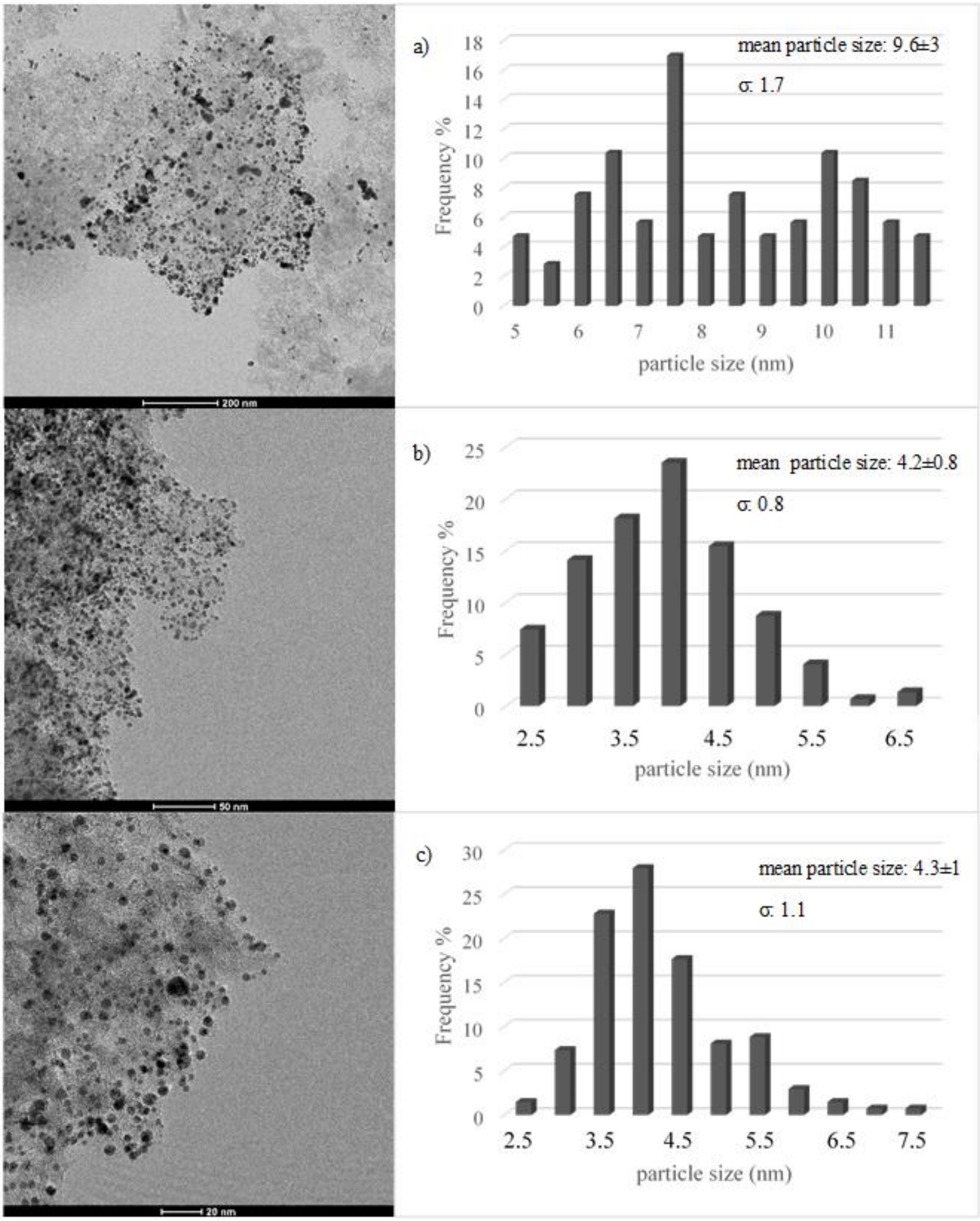
200 nm
a)
mean particle size: 9.6±3
σ: 1.7
Frequency %
0 2 4 6 8 10 12 14 16 18
5 6 7 8 9 10 11
particle size (nm)
50 nm
b)
mean particle size: 4.2±0.8
σ: 0.8
Frequency %
0 5 10 15 20 25
2.5 3.5 4.5 5.5 6.5
particle size (nm)
20 nm
c)
mean particle size: 4.3±1
σ: 1.1
Frequency %
0 5 10 15 20 25 30
2.5 3.5 4.5 5.5 6.5 7.5
particle size (nm)

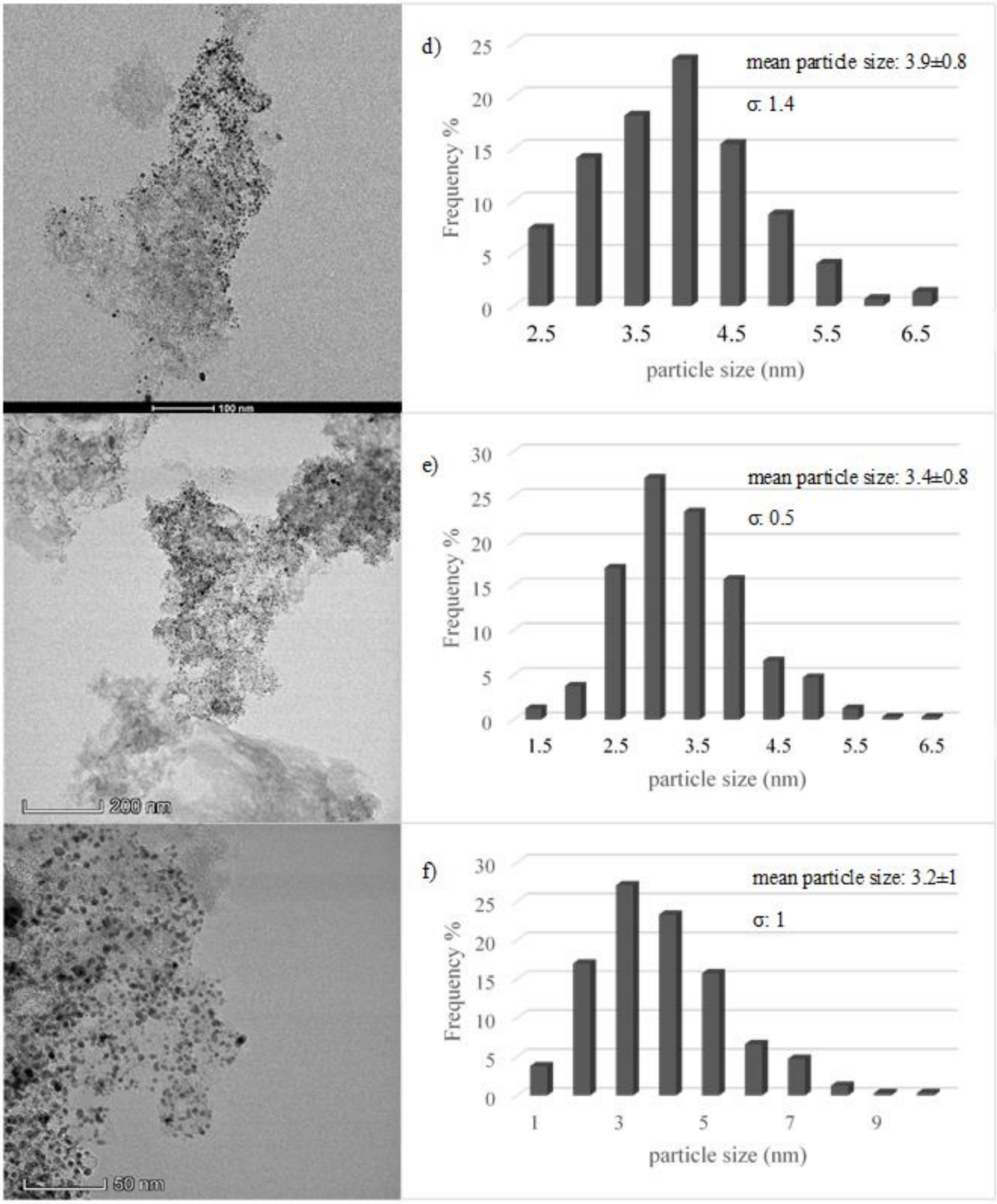

Figure S11. TEM images and particle size distributions of Au/AC synthesized using different hydrolysis degree: (**a**) 20%; (**b**) 40%; (**c**) 50%; (**d**) 60%; (**e**) 88% and (**f**) 99%.

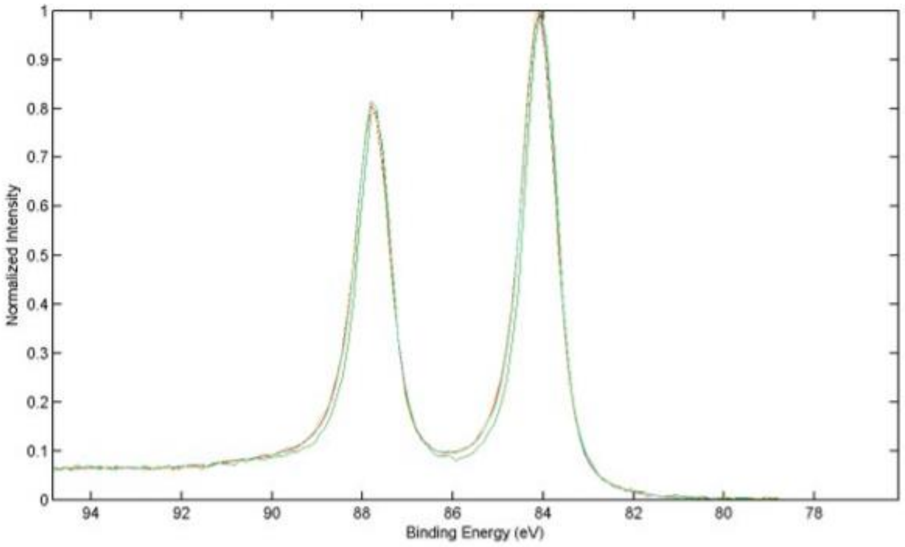

Figure S12. XPS spectra for 1% wt Au/AC synthesized using PVA (Mn:23,300) with different hydrolysis degree.

-Effect of molecular weight

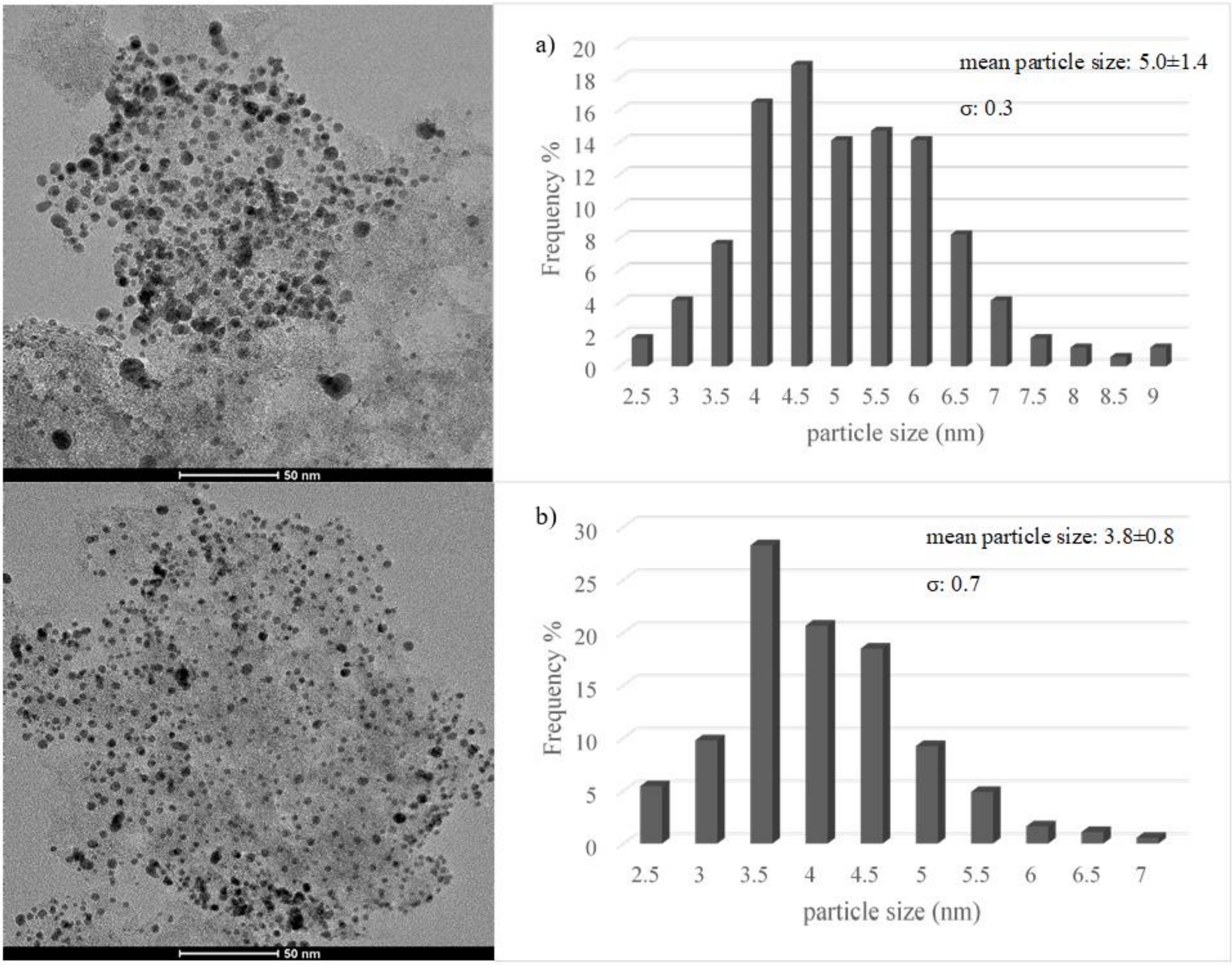

Figure 13. TEM images and particle size distributions of Au/AC synthesized using PVA2 (Mn=13,000–23,000) with different Au:PVA weight ratio: (**a**) ratio 1:0.15(Au:PVA); (**b**) ratio 1:0.33(Au:PVA).

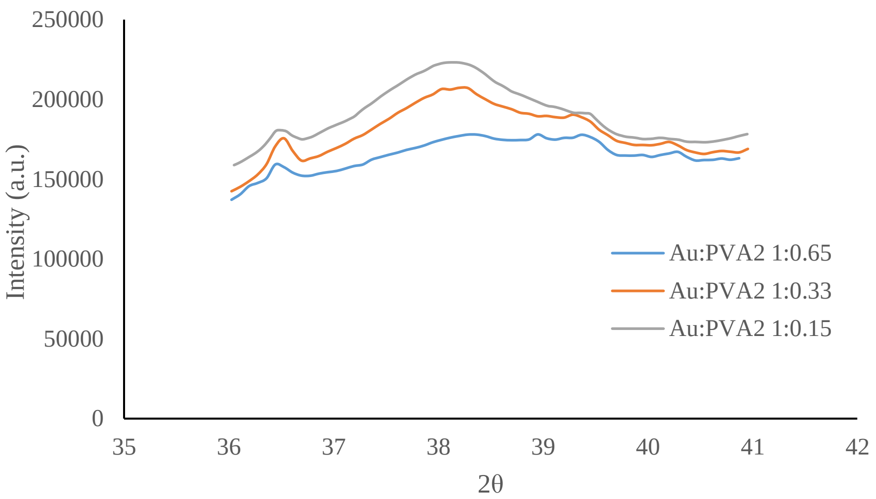

Figure S14. XRD patterns of the Au supported nanoparticles obtained by PVA2 (Mn:13,000–23,000) with different Au:PVA weight ratio.

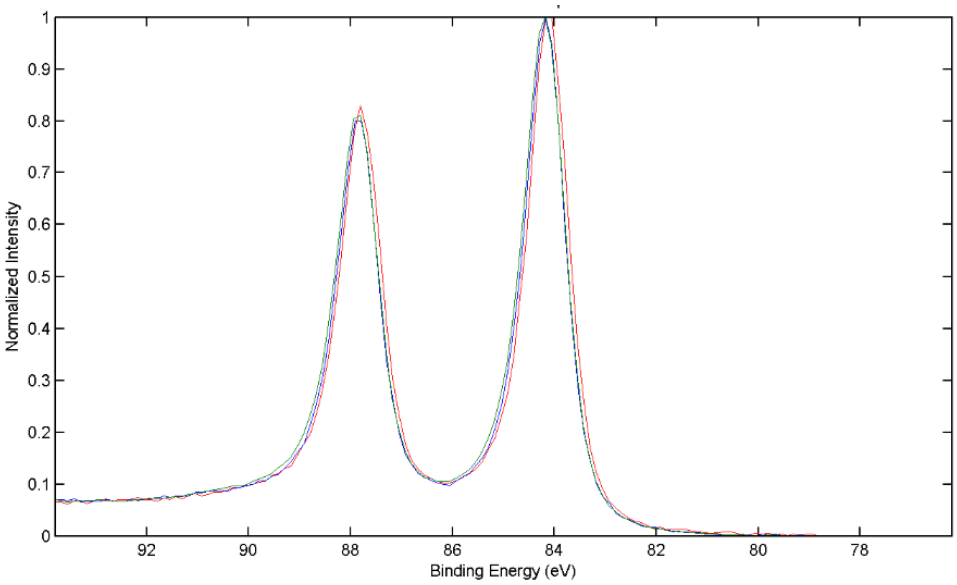

Figure S15. XPS spectra for 1% wt Au/AC synthesized using PVA2 (13,000–23,000) with different Au:PVA weight ratio.

-Effect of hydrolysis degree

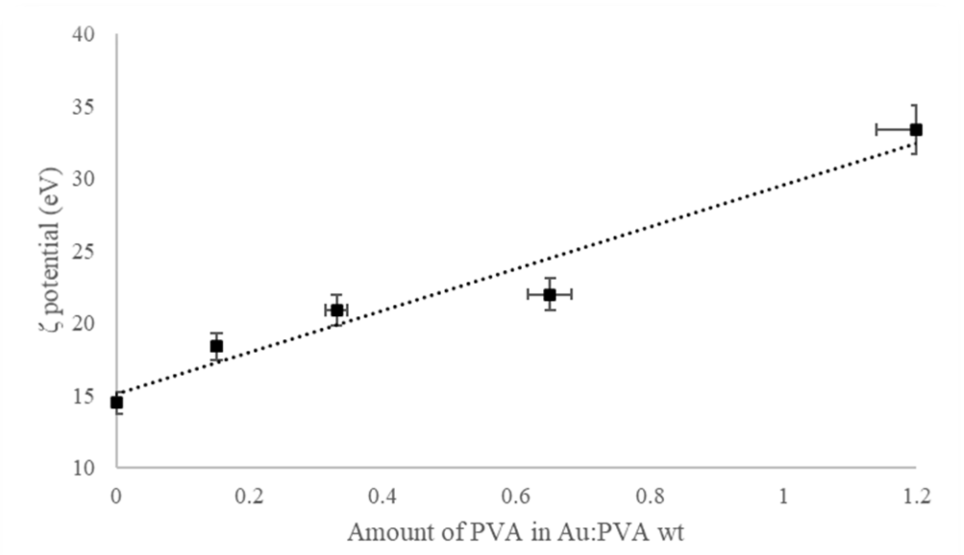

Figure S16. Zeta potential (ζ) of gold nanoparticles versus amount of PVA-60 (Mn:23,300) in Au:PVA weight ratio.

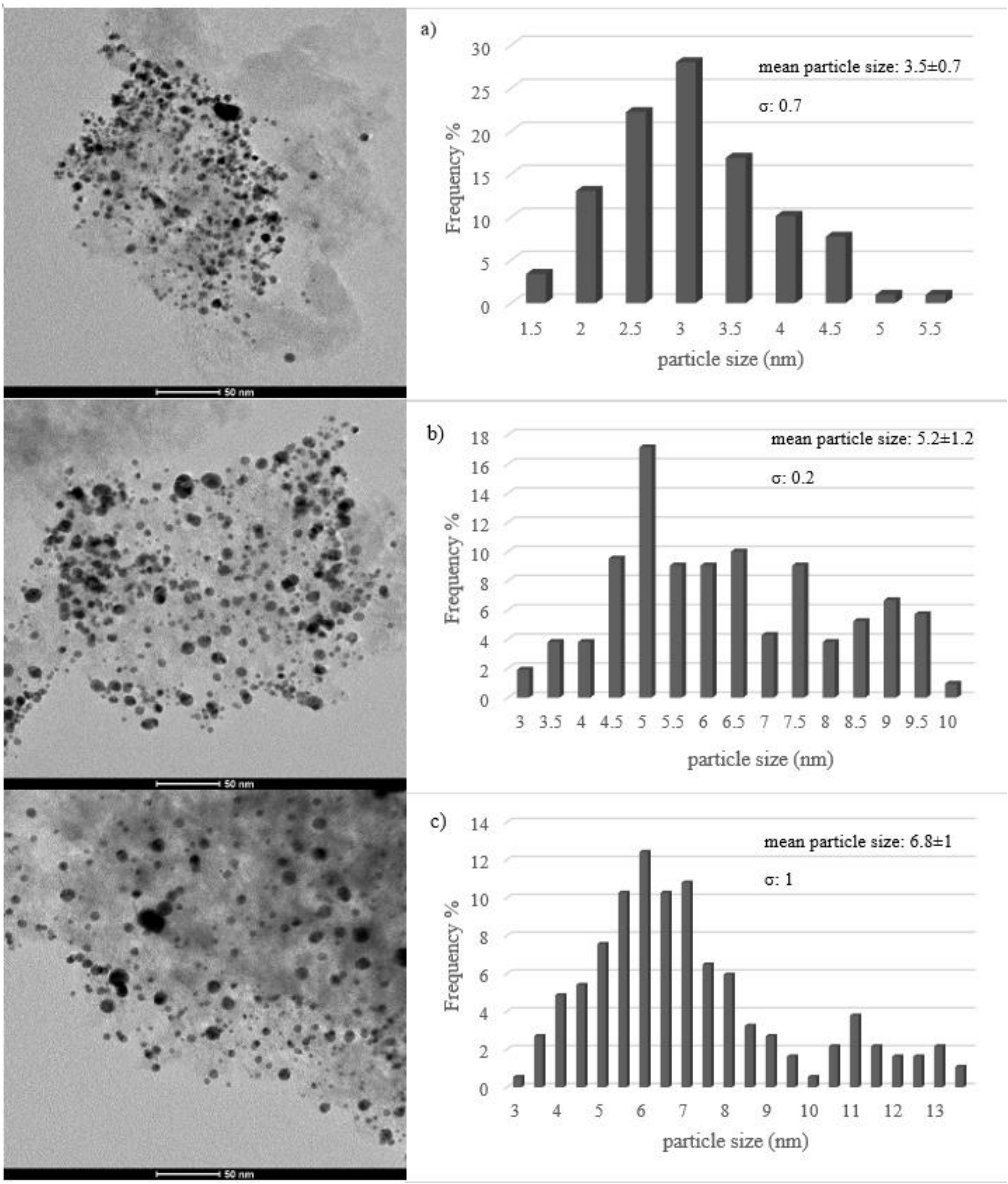
50 nm
a)
Frequency %
mean particle size: 3.5±0.7
σ: 0.7
0 5 10 15 20 25 30
1.5 2 2.5 3 3.5 4 4.5 5 5.5
particle size (nm)
50 nm
b)
Frequency %
mean particle size: 5.2±1.2
σ: 0.2
0 2 4 6 8 10 12 14 16 18
3 3.5 4 4.5 5 5.5 6 6.5 7 7.5 8 8.5 9 9.5 10
particle size (nm)
50 nm
c)
Frequency %
mean particle size: 6.8±1
σ: 1
0 2 4 6 8 10 12 14
3 4 5 6 7 8 9 10 11 12 13
particle size (nm)

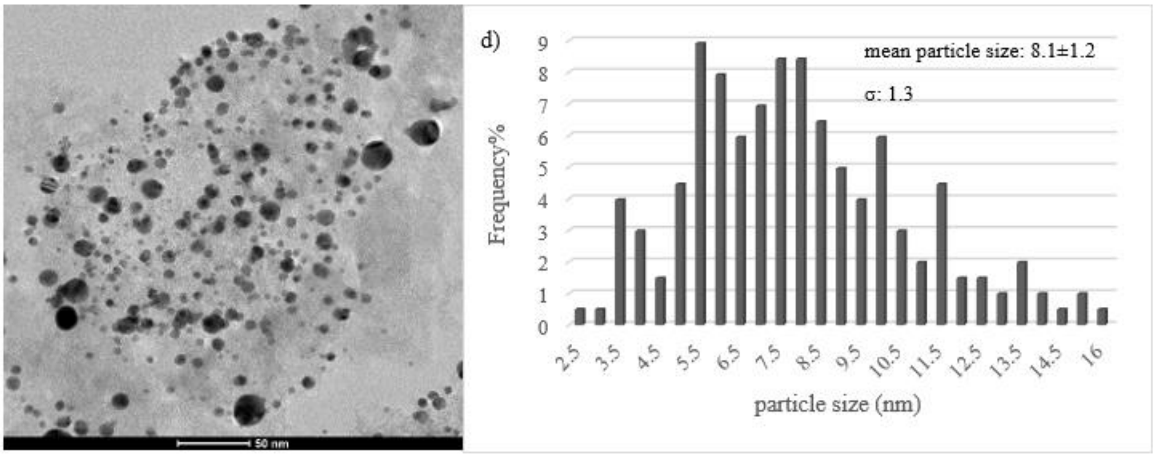

Figure S17. TEM images and particle size distributions of Au/AC synthesized using PVA-60 (Mn = 23,300) with different Au:PVA weight ratio: (**a**) ratio 1:1.2(Au:PVA); (**b**) ratio 1:0.33(Au:PVA); (**c**) 1:0.15 (Au:PVA); (**d**) 1:0 (Au:PVA).

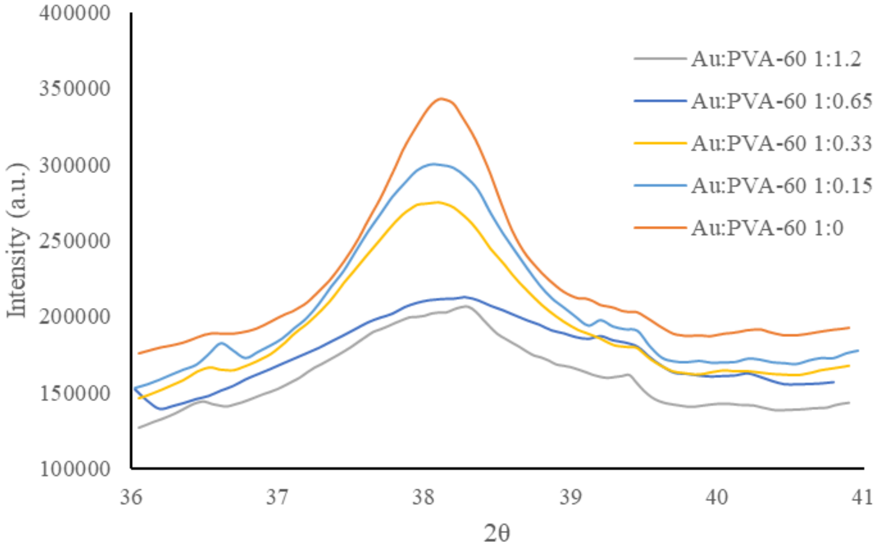

Figure S18. XRD patterns of the Au supported nanoparticles obtained by PVA-60 (Mn:23,300) with different Au:PVA weight ratio.

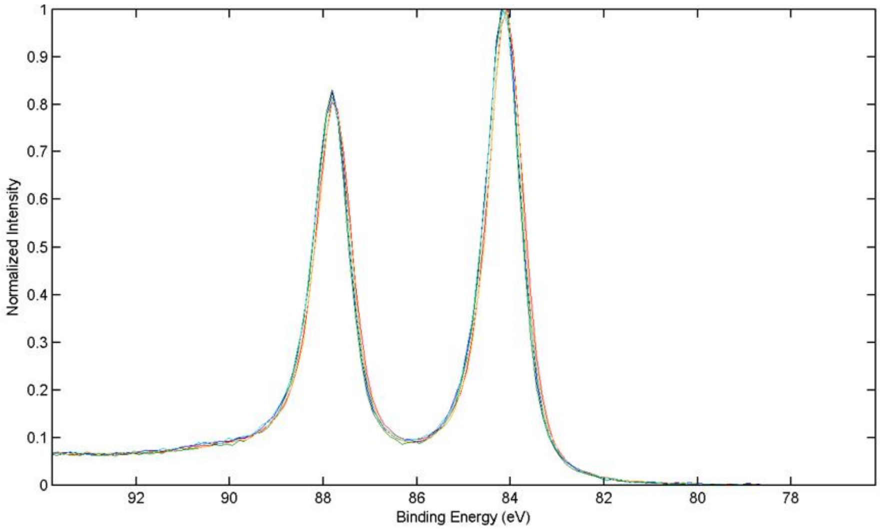

Figure S19. XPS spectra for 1% wt Au/AC synthesized using PVA-60 (23,000) with different Au:PVA weight ratio.

-Reusability test

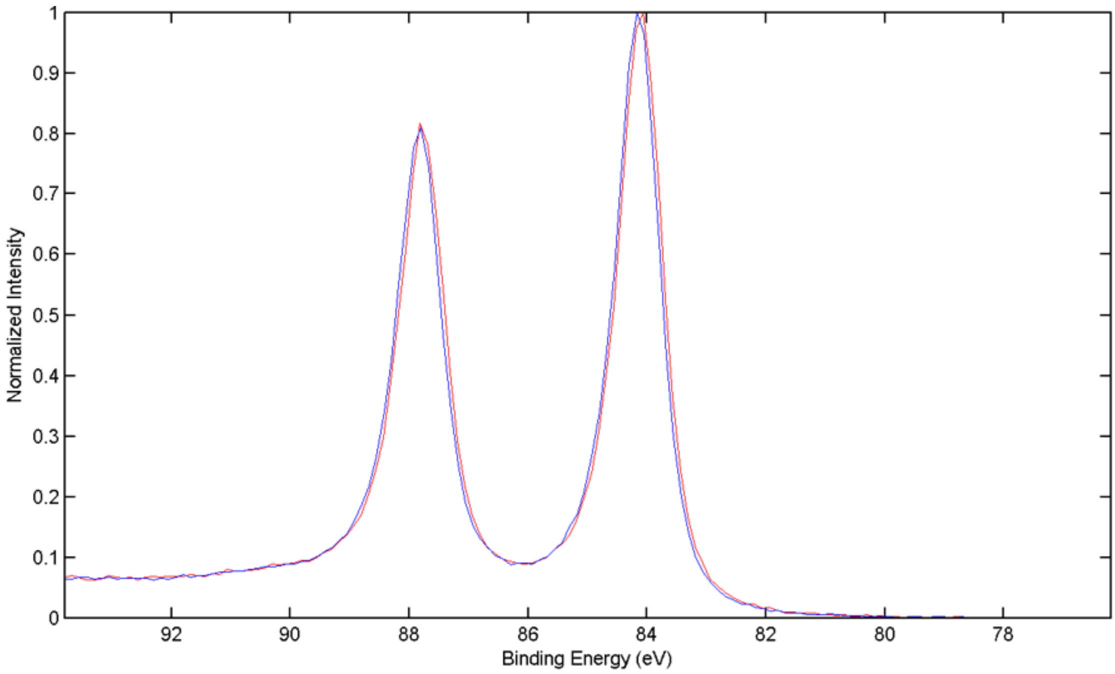

Figure S20. XPS spectra for 1% wt Au/AC synthesized using PVA-60 (23,000) with Au:PVA of 1:0.33: comparison beetween fresh and used catalyst.

Table S2. XPS data for 1% wt Au/AC synthesized using PVA-60 (Mn = 23,300) with Au:PVA of 1:0.33: comparison beetween fresh and used catalyst.

	**Au:PVA (w/w)**	*BE Au 4f7/2 (eV)*	*Au on Surface (% atomic)*	*C on Surface (% atomic)*	*Surface atomic ratio Au/C*
**PVA-60 fresh**	1:0.33	84.1	1.38	93.2	0.02
**PVA-60 used**	1.0.33	84.1	1.87	92.9	0.02

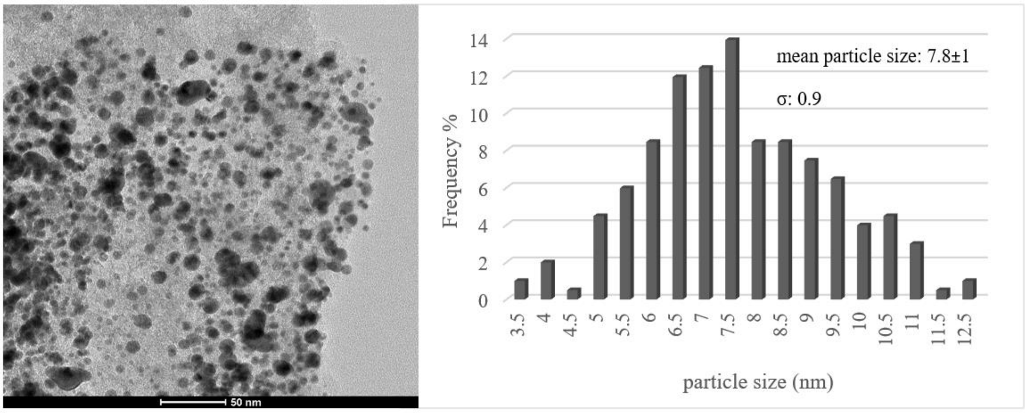

Figure S21. TEM images and particle size distributions of Au/AC used catalyst synthesized with PVA-60 (Mn = 23,300) Au:PVA of 1:0.33.
